# Identification of a BRAF/PA28γ/MEK1 signaling axis and its role in epithelial-mesenchymal transition in oral submucous fibrosis

**DOI:** 10.1038/s41419-022-05152-6

**Published:** 2022-08-12

**Authors:** Changqing Xie, Zaiye Li, Yufei Hua, Silu Sun, Liang Zhong, Qian Chen, Hui Feng, Ning Ji, Taiwen Li, Xikun Zhou, Xin Zeng, Zhangui Tang, Chongkui Sun, Jing Li, Qianming Chen

**Affiliations:** 1 State Key Laboratory of Oral Diseases, National Clinical Research Center for Oral Diseases, Chinese Academy of Medical Sciences Research Unit of Oral Carcinogenesis and Management, West China Hospital of Stom atology, Sichuan University, Chengdu, Sichuan 610041 People’s Republic of China; 2 Department of Oral and Maxillofacial Surgery, Xiangya Stomatological Hospital & School of Stomatology, Postdoctoral Research Workstation, Cancer Research Institute and School of Basic Medicine, Central South University, Changsha, Hunan 410078 People’s Republic of China; 3 State Key Laboratory of Biotherapy and Cancer Center, West China Hospital, Sichuan University and Collaborative Innovation Center for Biotherapy, Chengdu, Sichuan 610041 People’s Republic of China

**Keywords:** Mechanisms of disease, Oral cancer

## Abstract

Oral submucous fibrosis (OSF) is a chronic and insidious oral potentially malignant disorder associated with a 4–17% risk of oral squamous cell carcinoma (OSCC). Our previous study found that proteasomal activator 28 gamma (PA28γ) is frequently overexpressed in oral squamous cell carcinoma and negatively correlated with poor patient prognosis. However, the role of PA28γ in the occurrence and development of OSF remains unclear. Here, we screened PA28γ-related genes and investigated their function in OSF. We demonstrated that the expression of PA28γ was positively associated with MEK1 and gradually elevated from normal to progressive stages of OSF tissue. Arecoline, a pathogenic component of OSF, could upregulate the protein levels of PA28γ and phosphorylated MEK1 and contribute to epithelial to mesenchymal transition (EMT) in epithelial cells. Notably, PA28γ could interact with MEK1 and upregulate its phosphorylation level. Furthermore, arecoline upregulated BRAF, which can interact with PA28γ and upregulate its protein level. Additionally, BRAF, PA28γ, and MEK1 could form protein complexes and then enhance the MEK1/ERK signaling pathways. The concrete mechanism of the protein stability of PA28γ is that BRAF mediates its degradation by inhibiting its ubiquitination. These findings underscore the instrumental role of PA28γ in the BRAF/MEK1 pathway and enhanced EMT through MEK1/ERK activation in OSF.

## Introduction

Oral submucous fibrosis (OSF) is a chronic, inflammatory, and excessive tissue repair disease that is an insidious oral potentially malignant disorder (OPMD) [1, 2]. Clinically, the oral mucosa of OSF patients is replaced by irregular plate-like or leather-like fiber strips, resulting in restricted mouth opening, decreased taste, speech, and swallowing disorders [3]. It is estimated that approximately 7–14% of patients with OSF, especially those with hyperkeratosis, dysplasia, or keratinization, will progress to oral squamous cell carcinoma (OSCC) [4]. Areca nut chewing is the primary etiology of OSF occurrence and malignant transformation, and approximately one in ten of the world’s populations has the habit of chewing betel nut-related products, especially in some regions of South Asia, East Africa, and the tropical Pacific [5]. Among the areca alkaloids, arecoline is the main agent responsible for promoting the progression of OSF. As in previous studies, the concentration of arecoline in saliva fluctuates approximately 5–100 μg/mL during the process of betel nut chewing [6, 7].

Accumulating evidence has indicated that areca nut chewing is a major etiological factor that could induce epithelial to mesenchymal transition (EMT), which is a crucial process for carcinogenesis [8]. Numerous EMT transcription factors, such as ZEB-1 and Twist, were found to be involved in areca nut-associated OSF [9, 10]. The protein expression of EMT biomarkers, such as Vimentin and E-cadherin, are obviously increased or decreased, respectively [11, 12]. However, the molecular mechanisms that drive EMT in OSF epithelial cells remain unclear.

Proteasomal activator 28 gamma (PA28γ) was first identified as a Ki antigen, a nucleoprotein targeted by autoantibodies found in the serum of patients with systemic lupus erythematosus [13–15]. Structurally, PA28γ differs from its family proteins PA28α and PA28β, which have unique biological characteristics [16, 17]. In addition, PA28γ plays a crucial role in the cell cycle, cell proliferation, and anti-apoptosis processes, which are involved in the occurrence and development of tumors [18, 19]. Previous research identified that the expression level of PA28γ was upregulated in precancerous lesions, including oral leukoplakia (OLK) and oral lichen planus (OLP), and OSCC tissues with poor prognosis [20]. Moreover, PA28γ can enhance tumor cell migration and invasion and is associated with poor prognosis in OSCC [21, 22]. It has been suggested that OSF is one of the most potentially malignant oral disorders [4, 23]. In addition, the development of OSF involves numerous genetic variants and signaling pathways. Based on the above, we hypothesize that PA28γ and its related genes may contribute to the progression of OSF and its carcinogenesis.

Here, we demonstrated that PA28γ enhanced the differentiation of epithelial cells by promoting EMT in OSF. First, we found that the expression of PA28γ was positively associated with MEK1 and gradually elevated from normal to progressive stages of OSF tissue. Then, the pathogenic component of OSF, arecoline, was found to upregulate the protein levels of PA28γ and phosphorylated MEK1 and contribute to EMT in epithelial cells. Notably, PA28γ could interact with MEK1 and upregulate its phosphorylation level. Furthermore, arecoline upregulated BRAF, which can interact with PA28γ and upregulate its protein level. Additionally, BRAF, PA28γ and MEK1 could form protein complexes and then enhance the MEK1/ERK signaling pathways. The concrete mechanism of the protein stability of PA28γ is that BRAF mediates its degradation by inhibiting its ubiquitination. These findings underscore the instrumental role of PA28γ in the BRAF/MEK1 pathway and enhanced EMT through MEK1/ERK activation in OSF.

## Results

### The expression of PA28γ was positively associated with MEK1 and gradually elevated from normal to progressive stages of OSF tissue

**Fig. 1:**
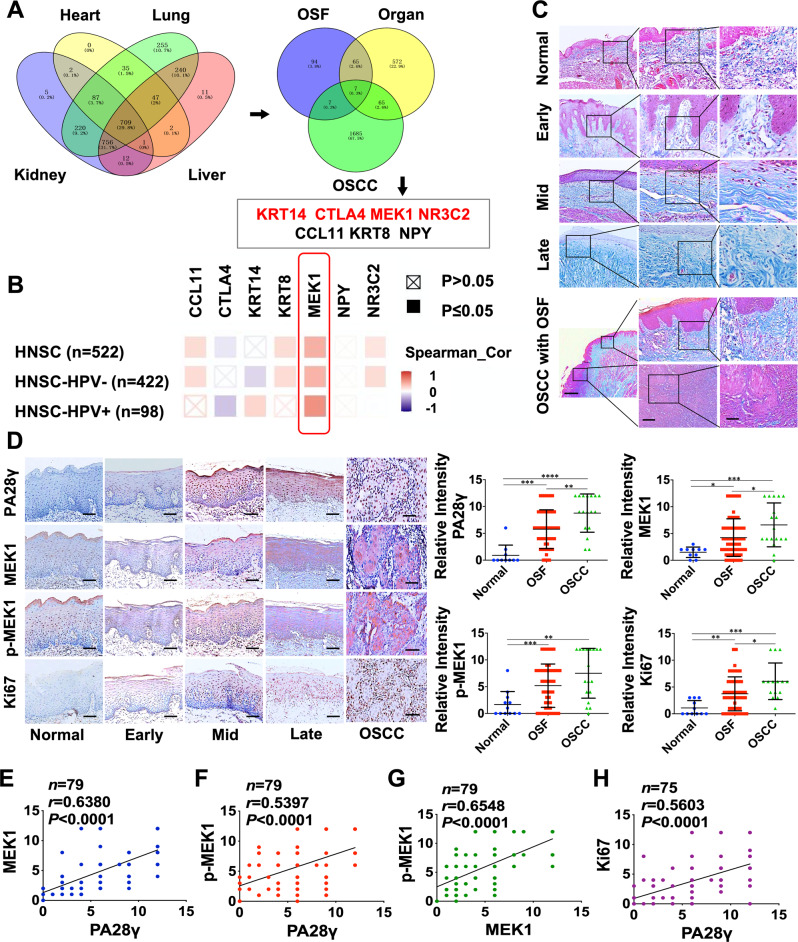
The expression of PA28γ in OSF epithelial tissue was positively correlated with both MEK1 and phosphorylated MEK1. **A** Obtaining OSF malignant transformation genes through database search (GeneCards) and bioinformatics analysis. A total of 709 organ fibrosis genes were associated with pulmonary fibrosis (2349), liver fibrosis (1778), renal fibrosis (1792) and myocardial fibrosis (883). A Venn diagram yielded 7 genes associated with organ fibrosis (709), OSF (181) and OSCC (1693). Among them, 4 upregulated genes (KRT14, CTLA4, MEK1, and NR3C2) and 3 downregulated genes (CCL11, KRT8, and NYP) were obtained. **B** Correlation analysis showed that the MEK1 gene positively correlated with PA28γ. **C** Masson’s trichrome staining for identifying different stages of OSF phenotype, including early, mid, late, and OSCC with OSF. Scale bar: left panels, 500 μm; mid panels, 200 μm; right panels, 100 μm. **D** IHC staining and statistical analysis showed the expression of PA28γ, MEK1, p-MEK1, and Ki67 in normal (*n* = 10), OSF (*n* = 52), and OSCC with OSF (*n* = 18) tissues. Based on Student’s t test (*p < 0.05, **p < 0.01, ***p < 0.001, ****p < 0.000). Scale bar: 200 μm. **E**–**H** Correlation analysis of 4 molecules in the normal, OSF and OSCC groups.

To screen the related genes of PA28γ and investigate their function in OSF, we first obtained reported gene sets that affect fibrosis in four organs. There were 709 common differentially expressed genes for these four types of organ fibrosis. We then used the intersection of these 709 genes, the gene sets that were differentially expressed in OSF, and the gene sets that were significantly related to the prognosis of OSCC patients to obtain 7 candidate genes, which included 4 upregulated (KRT14, CTLA4, MEK1, and NR3C2) and 3 downregulated (CCL11, KRT8, and NPY) genes (Fig. 1A). Subsequently, we used the data from TCGA database to analyze the correlation between these 7 candidate genes and the PA28γ coding gene. After combining the *P* value and the correlation coefficient, MEK1 was screened out (Fig. 1B). To explore the expression and correlation of PA28γ and MEK1 in the OSF cohort, we collected 52 OSF tissues, which included early-, mid- and late-stage samples, and 18 OSCC with OSF tissues. Masson’s trichrome staining showed that collagen deposition gradually became obvious with the development of OSF (Figs. 1C and S1). Immunohistochemical anti-PA28γ staining had almost no expression in normal control epithelium, and it showed weak cytoplasmic positivity and strong nuclear positivity in different stages of OSF epithelium. Relatively weak anti-MEK1 and anti-p-MEK1 immunoreactivity was detected in normal control epithelium, while both molecules had a similar intensity of PA28γ expression (Fig. 1D). In addition, the expression of Ki67 in the epithelium also had a marginal upward trend with the severity of fibrosis. Finally, the results of Pearson correlation analysis showed a dramatically positive correlation of protein levels between PA28γ and MEK1 (*r* = 0.6380, *p* < 0.0001), PA28γ and p-MEK1 (*r* = 0.5397, *p* < 0.0001), MEK1 and p-MEK1 (*r* = 0.6548, *p* < 0.0001), and PA28γ and Ki67 (*r* = 0.5603, *p* < 0.0001, Fig. 1E–H). Overall, the expression patterns of OSF clinical cohorts proved that PA28γ overexpression was correlated with MEK1 activity in OSF, suggesting that these molecules might participate in the development of OSF.

### Arecoline upregulated the protein levels of PA28γ and phosphorylated MEK1 and contributed to EMT in epithelial cells

**Fig. 2:**
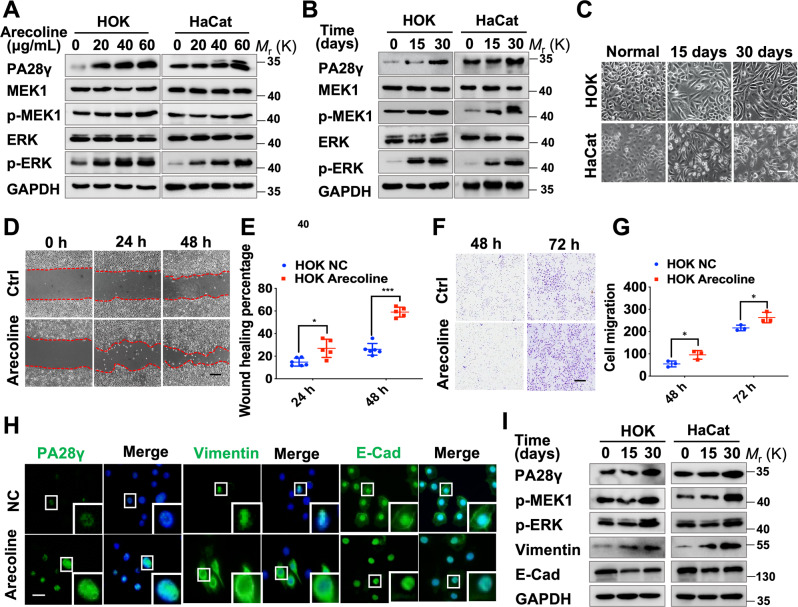
Arecoline can upregulate the expression levels of PA28γ and phosphorylated MEK1 in epithelial cells, which induce EMT. Western blot assays showed that arecoline upregulated the expression levels of PA28γ, p-MEK1, and p-ERK in an adosage-dependent (**A**) and time-dependent manner (**B**). **C** After 30 days of treatment of epithelial cells with low concentrations of arecoline (10 μg/mL), the morphology of epithelial cells transformed to a mesenchymal phenotype. Scale bar: 25 μm. Wound healing (**D**, **E**) and transwell assays **F**, **G** showed that the healing ability and migration ability of epithelial cells treated with arecoline were upregulated. Scale bar: 100 μm. Data represent the means ± s.d. of three independent experiments. Statistical analysis was performed using Student’s t test (*p < 0.05, ***p < 0.001). **H** Representative results for immunostaining of PA28γ, Vimentin, and E-cadherin in the normal control group and the arecoline-treated group. Scale bar: 50 μm. **I** Western blot assays showed the expression levels of Vimentin and E-cadherin in arecoline-treated epithelial cells.

Arecoline is the major areca nut alkaloid and has been classified as carcinogenic to humans; it is also considered the main causative factor of OSF due to betel nut chewing [24]. To explore the function and relationship between PA28γ and MEK1 in vivo. First, we treated epithelial cells with arecoline to simulate the state of epithelial cells in OSF. The concentration range of 20–60 µg/mL effectively inhibited epithelial cell proliferation activity in vitro (Fig. S2A, B), and the mRNA expression of PA28γ and MEK1 was slightly upregulated in HaCat cells but not in HOK cells (Fig. S2C–F). Then, we explored whether arecoline affects the protein expression levels of PA28γ and MEK1. The results showed that the expression levels of PA28γ, p-MEK1, and p-ERK, which are downstream of MEK1, were significantly upregulated in a dosage-dependent or a time-dependent manner after arecoline treatment in both epithelial cell lines (Fig. 2A, B). Immunofluorescence staining also confirmed that arecoline could induce the expression levels of PA28γ and p-MEK1 in epithelial cells (Fig. S3). Interestingly, after prolonged stimulation of epithelial cells with low concentrations of arecoline, we found that epithelial cell morphology gradually evolved from a polygonal phenotype to a spindle phenotype (Fig. 2C). Simultaneously, the migration ability of epithelial cells treated with arecoline was significantly enhanced (Fig. 2D–G). Subsequently, the protein expression of the EMT biomarkers Vimentin and E-cadherin was obviously increased or decreased, respectively, in the arecoline treatment group compared with the control group (Fig. 2H, I).

In addition, we constructed an OSF mouse model induced by arecoline and bleomycin as a positive control (Fig. S4A) [25]. Compared with the PBS group, Masson’s trichrome staining in the arecoline or bleomycin group showed collagen accumulation in the lamina propria, and the blood vessels were significantly reduced (Fig. S4B), suggesting successful modeling. Immunohistochemical staining showed that arecoline could induce the expression of PA28γ in the mouse buccal epithelium. Correspondingly, the expression levels of N-cadherin and Vimentin were upregulated, while the expression level of E-cadherin was downregulated (Fig. S4C). In general, arecoline stimulation upregulated the protein levels of PA28γ and phosphorylated MEK1 and induced EMT in epithelial cells.

### PA28γ interacts with MEK1 and upregulates its phosphorylation level

**Fig. 3:**
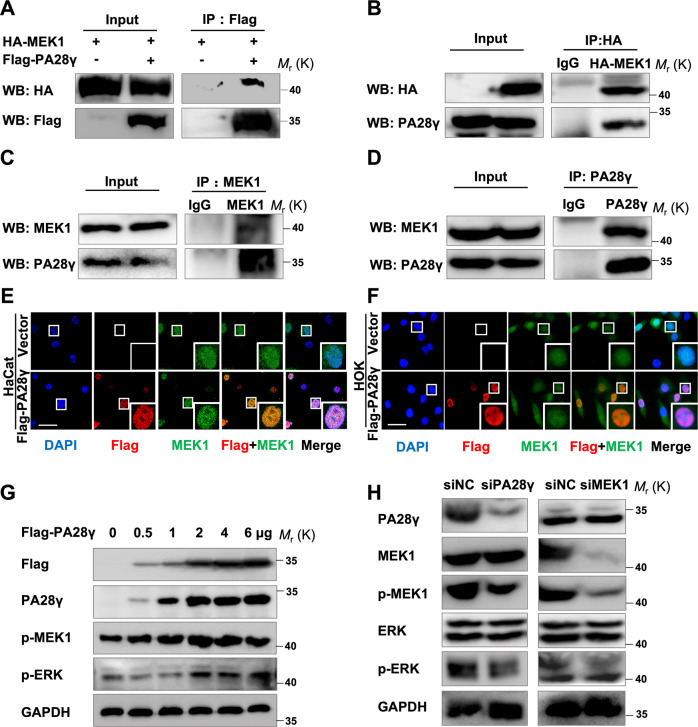
PA28γ can bind to MEK1 and activate the MEK1/ERK signaling pathway. **A**, **B** HA-tagged MEK1 and Flag-tagged PA28γ plasmids were transfected into 293T cells, and immunoprecipitation experiments showed that PA28γ and MEK1 have a direct interaction relationship. Immunoprecipitation of MEK1 was performed with the HA-MEK1 antibody. **C**, **D** Coimmunoprecipitation assays showed that endogenous PA28γ and endogenous MEK1 could bind to each other. Immunoprecipitation of PA28γ was performed with the anti-PA28γ or anti-Flag antibody. **E**, **F** Representative results of the coimmunostaining of Flag-PA28γ (red) and endogenous MEK1 (green) in epithelial cells. The nuclei were stained blue with DAPI. Scale bar: 50 μm. **G** After the Flag-tagged PA28γ plasmid was transfected into 293T cells in a dose-dependent manner, western blot assays showed that the expression levels of p-MEK1 and p-ERK were upregulated. **H** Western blot assays showed that the epithelial cells were treated with siRNA-PA28γ, siRNA-MEK1, or siNC.

To detect whether there is a direct interaction between PA28γ and MEK1, which have the same expression trend in OSF tissue and arecoline-treated epithelial cells, we constructed HA-MEK1 and Flag-PA28γ plasmids. Flag preset beads could pull down HA-MEK1, and HA antibody could also immunoprecipitate Flag-PA28γ in cells transfected with HA-tagged MEK1 and Flag-tagged PA28γ plasmids (Fig. 3A, B). Coimmunoprecipitation analysis showed that both endogenous PA28γ and MEK1 could bind to each other (Fig. 3C, D). Immunofluorescence analyses with anti-Flag or anti-MEK1 antibodies showed that Flag-PA28γ was mainly located in the nucleus and showed significant nuclear localization and clear colocalization with MEK1 in HOK and HaCat cells (Fig. 3E, F). Then, we found that PA28γ could upregulate MEK1 phosphorylation levels in a dose-dependent manner, and the protein levels of p-ERK, which is the downstream molecule of p-MEK1, were also upregulated. However, when MEK1 was upregulated, there was no effect on the protein level of PA28γ (Figs. 3G, S5A, B). The PA28γ-dependent increase in p-MEK1 was restored by transfection with siRNA-PA28γ, and the protein level of PA28γ had no obvious effect when siRNA-MEK1 appeared (Fig. 3H). These data suggested that PA28γ could interact with MEK1 and upregulate its phosphorylation level and that MEK1 may be downstream of PA28γ. In addition, overexpressing PA28γ induced the translocation of a large portion of p-MEK1 to the nucleus and around the nucleus in two epithelial cell lines (Fig. S5C, D). This evidence suggests that PA28γ could interact with MEK1 and upregulate its phosphorylation level.

### PA28γ-dependent MEK1 upregulation contributes to EMT of epithelial cells

**Fig. 4:**
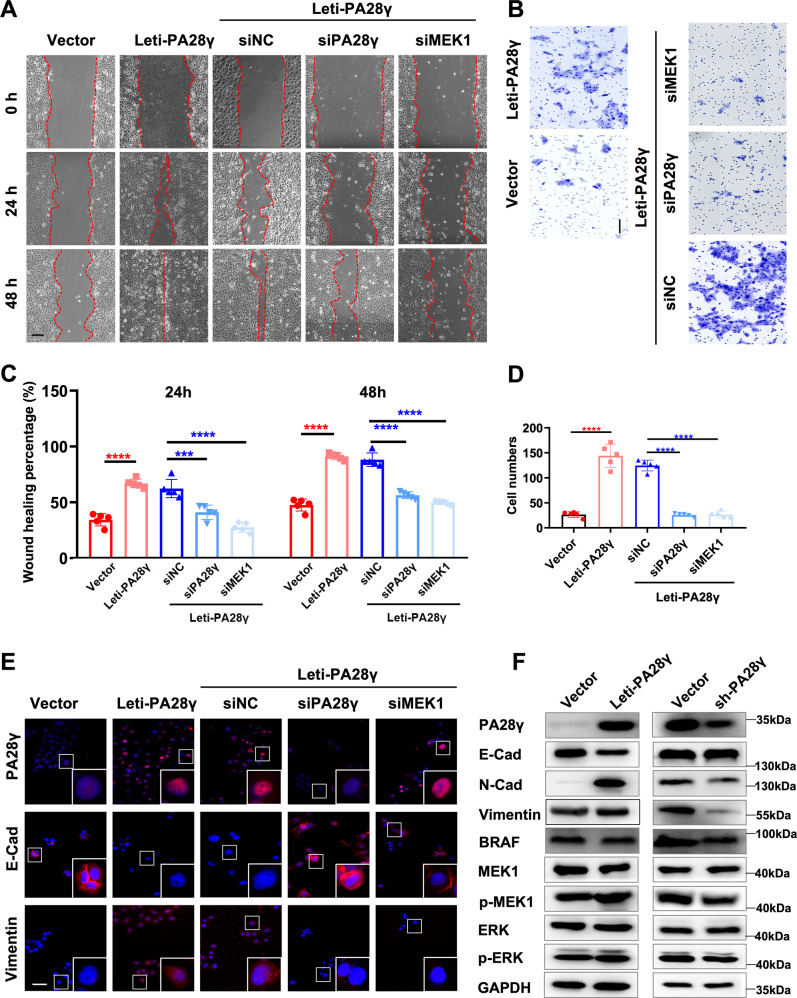
PA28γ-dependent upregulation of phosphorylated MEK1 promotes EMT in epithelial cells. **A**, **B** Wound healing and transwell assays showed that epithelial cells overexpressing PA28γ by lentivirus acquired enhanced migration capacity, which could be reduced by knocking down PA28γ or MEK1 expression. Scale bar: 100 μm. **C**, **D** Data represent the means ± s.d. of three independent experiments. Statistical analysis was performed using Student’s t test (***p < 0.001, ****p < 0.000). **E** Immunostaining and **F** western blot assays showed that overexpressed PA28γ led cells to acquire a mesenchymal phenotype with higher Vimentin expression and lower E-cadherin expression. Knocking down MEK1 in epithelial cells stably overexpressing PA28γ partly reversed the EMT phenotype. Scale bar: 50 μm.

To determine where PA28γ-dependent MEK1 upregulation contributes to EMT of epithelial cells, we employed a series of cell biology experiments. As a result, overexpressing PA28γ enhanced epithelial cell migration, which could be reversed by siRNA. We knocked down MEK1 in epithelial cells stably overexpressing PA28γ, which reduced the migration capacity of cells. In parallel, when an inhibitor of MEK1, CI1040, was applied to treat epithelial cells stably overexpressing PA28γ, similar results were obtained (Figs. 4A–D, S6A, B).

Because EMT plays an important role in the carcinogenesis of OSF, we next detected EMT biomarker expression in cells with different PA28γ and MEK1 expression levels. As a result, higher PA28γ led cells to transform to a mesenchymal phenotype while losing epithelial biomarkers. Knocking down or inhibiting MEK1 in epithelial cells stably overexpressing PA28γ partly reversed the EMT phenotype (Figs. 4E, F, S6C). These data suggest that PA28γ contributes to the EMT of epithelial cells by activating MEK1.

### Arecoline-dependent upregulation of BRAF interacts with PA28γ and upregulates its protein level

**Fig. 5:**
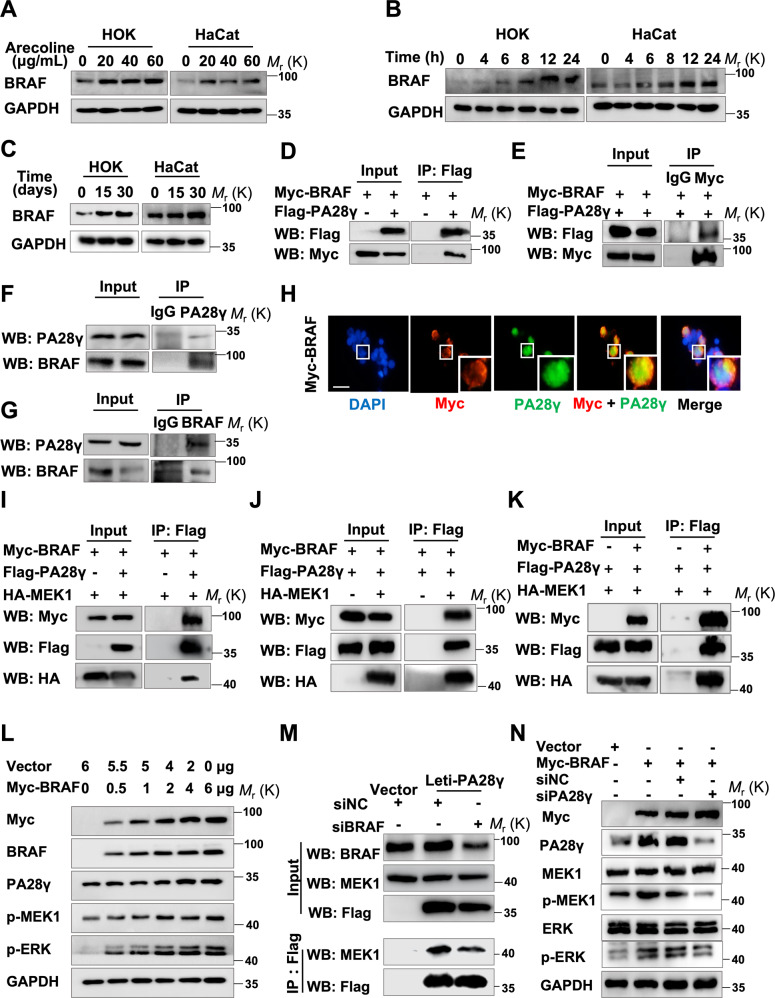
BRAF can bind to PA28γ and stabilize its protein level. **A**–**C** Western blot assays showed that the expression levels of BRAF were upregulated in a dosage-dependent and time-dependent manner. **D**, **E** A coimmunoprecipitation assay showed that exogenous Myc-BRAF and Flag-PA28γ bind to each other in 293T cells. **F**, **G** Coimmunoprecipitation experiments showed that endogenous BRAF and PA28γ interact with each other in epithelial cells. **H** After transfecting the Myc-BRAF plasmid into 293T cells, coimmunostaining showed that Myc-BRAF (red) colocalized with PA28γ (green). Scale bar: 50 μm. **I** Immunoprecipitation assays showed that exogenous BRAF and MEK could simultaneously bind to PA28γ. Immunoprecipitation of PA28γ was performed with the Flag antibody. **J** Exogenous BRAF and PA28γ were detected in immunoprecipitates performed with the HA antibody. **K** Exogenous MEK1 and PA28γ can be detected in immunoprecipitates performed with the Myc antibody. **L** Transfection of 293T cells with the Myc-BRAF plasmid in a dose-dependent manner. Western blot assays showed that the expression levels of PA28γ, p-MEK1, and p-ERK were significantly upregulated. **M** Immunoprecipitation shows that silencing BRAF expression reduces MEK1 binding to PA28γ. **N** Western blot assays showed that knocking down PA28γ inhibited BRAF-dependent expression of p-MEK1 and p-ERK.

PA28γ has been reported to bind to the N-terminal regulatory domain of MEKK3, a MAPK kinase kinase, which phosphorylates and raises the protein level of PA28γ [26]. BRAF is a well-known upstream kinase of MEK1, which belongs to the homolog of the MEKK3 family [27, 28]. Notably, the expression level of BRAF was significantly upregulated in a dosage- and time-dependent manner after arecoline treatment in epithelial cells (Fig. 5A–C), similar to PA28γ and p-MEK1. We, therefore, explored the relationship between BRAF and PA28γ. Coimmunoprecipitation analysis showed that exogenous PA28γ could pull down exogenous BRAF, and exogenous BRAF could also pull down exogenous PA28γ (Fig. 5D, E). Moreover, endogenous BRAF and endogenous PA28γ could bind to each other (Fig. 5F, G). In line with these findings, IF analyses showed that BRAF colocalized with PA28γ (Fig. 5H). These results strongly suggest that BRAF interacts with PA28γ. To further evaluate the relationship between BRAF, PA28γ, and MEK1, we performed coimmunoprecipitation analyses, which showed that the complex of BRAF, MEK, and PA28γ could be detected by immunoprecipitation (Fig. 5I–K). In addition, the protein levels of PA28γ were upregulated in a BRAF protein concentration-dependent manner, and the protein levels of p-MEK1 and p-ERK also showed the same trend (Figs. 5L, S7A). In turn, the protein level of BRAF showed no effect when PA28γ was overexpressed (Fig. S7B). Intriguingly, after disturbing the expression of BRAF in stable PA28γ-overexpressing epithelial cell lines, the expression of MEK1 was significantly reduced in PA28γ immunoprecipitates, suggesting that blocking BRAF expression could prevent the interaction between PA28γ and MEK1 (Fig. 5M). Consistently, the phosphorylation levels of MEK1 and ERK induced by PA28γ were also reversed (Fig. S7C). Finally, depletion of PA28γ inhibited BRAF-dependent expression of p-MEK1 and p-ERK but had no effect on BRAF expression, which indicated that PA28γ was a substrate of BRAF (Fig. 5N). Collectively, these cellular experiments illustrated that arecoline could upregulate BRAF and that BRAF can form protein complexes with PA28γ and MEK1 and then enhance the MEK1/ERK signaling pathways.

### The stability of the PA28γ protein is mediated by BRAF inhibiting its ubiquitination

**Fig. 6:**
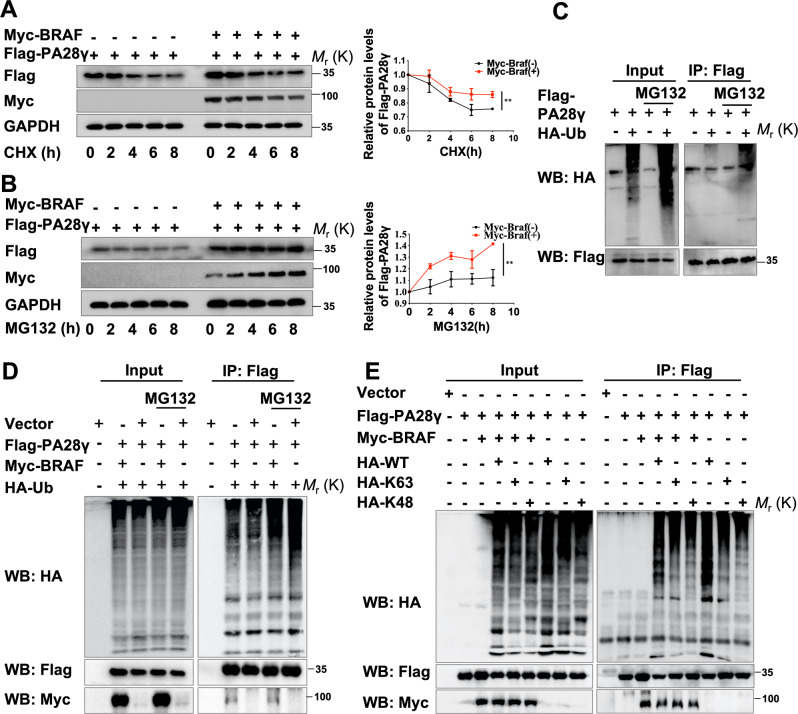
BRAF inhibited the ubiquitination level of PA28γ. 293T cells were constantly transfected with vector (2 μg), Flag-PA28γ (2 μg), or Myc-BRAF plasmid (2 μg) 24 h later, and then serum-starved 293T cells were pretreated with **A** CHX (100 μg/ml) or **B** MG132 (10 μM) for the indicated periods of time. Data represent the means ± s.d. of three independent experiments. Quantification of Flag-PA28γ levels relative to GAPDH is shown. Statistical analysis was performed using Student’s t test (**p < 0.01). **C** The ubiquitylation assay showed the ubiquitination degradation process of the PA28γ protein. **D**, **E** BRAF inhibited the PA28γ ubiquitination level at both the K48 and K63 sites.

To demonstrate the specific molecular mechanism underlying BRAF-regulated PA28γ upregulation, we pretreated 293T cells with cycloheximide (CHX) to block protein synthesis and detected the degradation of PA28γ protein. We found that this treatment had a limited effect on PA28γ degradation when BRAF appeared (Fig. 6A). Consistent with this result, when 293T cells were treated with the proteasome inhibitor MG123, the protein synthesis rate of PA28γ was significantly increased after BRAF treatment (Fig. 6B). The above experimental results suggest that BRAF enhances expression primarily by enhancing PA28γ stability. To determine whether BRAF stabilizes the protein level of PA28γ by inhibiting its ubiquitination degradation, we performed a ubiquitination assay and showed that the PA28γ protein has a ubiquitination degradation process (Fig. 6C). As expected, BRAF inhibited the ubiquitination level of PA28γ in a manner partially dependent on the K48 site and partially dependent on the K63 site (Fig. 6D, E). In addition, arecoline inhibited the ubiquitination level of the PA28γ protein (Fig. S8), suggesting that arecoline may inhibit PA28γ ubiquitination mediated by BRAF to stabilize its protein level.

## Discussion

**Fig. 7:**
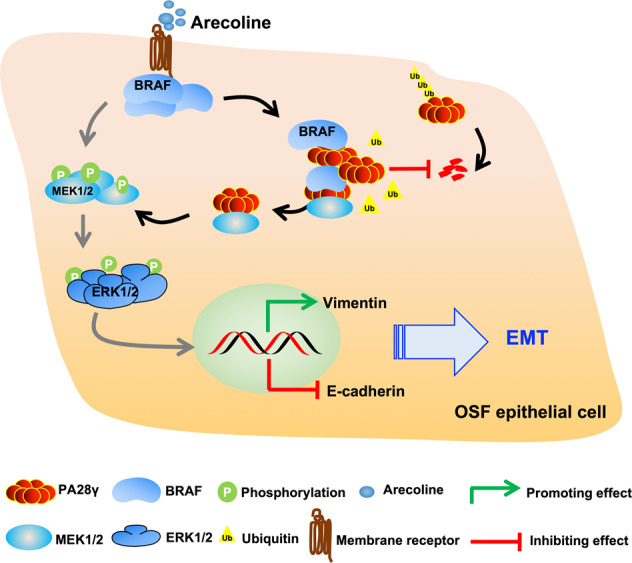
The pattern diagram of the BRAF/PA28γ/MEK1 signaling axis and its role in epithelial-mesenchymal transition in OSF. In brief, arecoline could induce BRAF upregulation and subsequent aberrant accumulation of PA28γ protein, which resulted in BRAF-mediated ubiquitination inhibition, leading to activation of MEK, and MEK-dependent activated ERK amplified the MAPK cascade and ultimately promoted EMT in epithelial cells.

OSF is a potentially malignant disease of the oral cavity that was listed as a precancerous state of the oral mucosa by the World Health Organization (WHO) in 1978 [29]. It is estimated that approximately 7–14% of patients with OSF will progress to OSCC. However, the molecular mechanism remains unclear. Therefore, exploring the key regulatory molecules and mechanisms in the progression of OSF could be beneficial to prevent the occurrence and block malignant transformation. Excitingly, we found solid evidence that arecoline, a pathogenic component of OSF, could induce BRAF upregulation and subsequent aberrant accumulation of PA28γ protein, which resulted in BRAF-mediated ubiquitination inhibition, leading to activation of MEK1. MEK1-dependent activated ERK phosphorylation amplified the MAPK cascade and ultimately promoted EMT in epithelial cells (Fig. 7). Our study highlights the underlying mechanism by which the BRAF/PA28γ/MEK1 axis enhances the differentiation of epithelial cells by promoting EMT in OSF.

Evidence suggests that PA28γ plays an important role in carcinogenesis [14, 15]. Our previous studies have clarified that PA28γ is closely related to the carcinogenesis of oral mucosa, including oral leukoplakia and OLP, and its high expression is associated with poor patient prognosis of OSCC [21, 30]. To further reveal the role and molecular mechanism of PA28γ in OSF, we used online database analysis to identify MEK1 as a gene positively correlated with the PA28γ signature in OSF. Subsequently, we confirmed that the expression of PA28γ was positively associated with MEK1 and gradually elevated from normal to progressive stages of OSF tissue, suggesting that these molecules might participate in the development of OSF.

A great deal of studies have shown that the development of OSF has been intimated to chewing betel nut commodity [31]. Arecoline is the major areca nut alkaloid and has been classified as carcinogenic to humans; it is also considered the main causative factor of OSF due to chewing betel nut [32, 33]. However, how arecoline promotes OSF progression is unclear. Here, we found that arecoline could upregulate the protein levels of PA28γ and phosphorylated MEK1. EMT refers to the biological process by which epithelial cells undergo multistep and complex biochemical changes to obtain the phenotype of mesenchymal cells [34]. Under normal physiological conditions, such as wound healing, proper EMT is necessary for the remodeling of the epithelial structure and the deposition of extracellular matrix, but continuous EMT may lead to the occurrence of various fibrotic diseases and cancer metastasis [35]. We found that prolonged treatment with arecoline in epithelial cells induced a spindle-shaped EMT phenotype, which, with decreased expression of the cell adhesion molecule E-cadherin, enhanced Vimentin-based cytoskeleton transformation. This result is consistent with published articles, which showed that arecoline can induce the mesenchymal differentiation of different types of cells, including epithelial cells, fibroblasts, and endothelial cells, to promote the occurrence of OSF and malignant transformation [11, 36]. Additionally, the wound healing and migration abilities of epithelial cells were significantly increased after arecoline treatment. In terms of the molecular mechanism, inhibiting the PA28γ/MEK1 pathway can downregulate the expression of Vimentin and upregulate the expression of E-cadherin, ultimately reversing the migration ability of epithelial cells. Previously, it was reported that PA28γ could promote the degradation of GSK-3β, leading to the accumulation of β-catenin and activation of the Wnt signaling pathway in skin carcinogenesis [37]. Wnt is an illustrious transcription factor of EMT, and these data corroborate our results.

MEK1 is an important key signaling molecule in the RAF/MEK/ERK pathway [28, 38]. In the occurrence of a variety of tumors, the continuous activation of MEK drives the uncontrolled proliferation and survival of tumor cells [39, 40]. The MEK1 protein is relatively conserved, and there is a nuclear export signal (NES) between the N-terminal D domain and the catalytic core, which is a key domain for MEK function and cellular positioning [41, 42]. We found that PA28γ can interact with MEK1 to activate the phosphorylation of MEK1 and promote the translocation of its nucleus to the cytoplasm. This result is likely caused by PA28γ affecting the amino acid sequence of the NES region. Regrettably, our study only provided some initial evidence about the underlying mechanism. In future work, we will explore the interaction sites of PA28γ and MEK1, as well as the biological functions of the two molecules after the interaction.

Posttranslational modifications (PTMs) refer to chemical modifications during or after protein translation [43]. PTMs play an irreplaceable role in a variety of cellular processes, such as protein interactions, protein breakdown, signal transduction, and gene expression regulation [44, 45]. In eukaryotes, PA28γ has a variety of PTM processes. For example, mutants of amino acids at different SUMO sites of PA28γ can be constructed, which can make PA28γ chemotactic to the cytoplasm and ultimately maintain its own protein stability [46, 47]. In addition, the yeast two-hybrid experiment showed that MEKK3 can interact with PA28γ. Then, the GST pull-down experiment showed that the binding of PA28γ and MEKK3 occurred in the N-terminal regulatory domain of MEKK3, leading to phosphorylation of PA28γ in vitro [26, 48]. Interestingly, we found that PA28γ interacts with the MEK1 upstream protein BRAF in vivo, which is a homolog of MEKK3. More importantly, coimmunoprecipitation experiments showed that BRAF, PA28γ, and MEK1 can form a protein complex. Additionally, a ubiquitination assay found that BRAF inhibits the PA28γ ubiquitination level and protects it from degradation. RAF/MEK/ERK, based on in-depth research in recent decades, has become a classic tumor signaling pathway that is closely related to the occurrence of multiple cancers. Taken together, our data strongly suggest that PA28γ is involved in the regulation of MEK by BRAF and that the BRAF/PA28γ/MEK1 signaling axis is involved in OSF.

In summary, our findings delineate PA28γ as an essential regulatory factor in OSF epithelial cell differentiation. A possible mechanism is that high concentrations of arecoline upregulate the expression level of BRAF in epithelial cells during the process of chewing betel nut and subsequent aberrant accumulation of PA28γ protein, which results in BRAF-mediated ubiquitination inhibition, leading to activation of MEK. MEK-dependent activated ERK phosphorylation amplifies the MAPK cascade and ultimately promotes EMT of epithelial cells. These findings underscore the instrumental role of PA28γ in the BRAF/MEK1 pathway and enhanced EMT through MEK1/ERK activation in OSF.

## Materials and methods

### Patient tissue samples

The study protocol was conducted in accordance with the principles of the Declaration of Helsinki and was approved by the Research Ethics Board of the Scientific and Ethical Committee of Xiangya Stomatological Hospital (20210018). And contents were obtained from all patients. A total of 52 OSF and 18 OSCC with OSF paraffin-embedded samples were obtained from Xiangya Stomatological Hospital, Central South University, Hunan, China. OSF specimens were derived from the pathological biopsy tissue of the patient’s first visit, and OSCC specimens were derived from the pathologically resected tissue of the patient after surgery. Ten normal tissues were resected from the oral mucosa of healthy people during oral surgery outpatient surgery (Table S1). The final diagnoses by two experienced pathologists were recruited for this study.

### Immunohistochemistry

Immunohistochemical staining of PA28γ, MEK1, p-MEK1, and Ki67 was performed following the protocols of previous reports. Paraffin-embedded sample sections were cut at 4 μm thickness, deparaffinized by heating at 60–65 °C (approximately 30 min), deparaffinized in xylene, and hydrated. Heat-mediated antigen retrieval was performed (5 min), and sections were immersed in 3% hydrogen peroxide for 15 min to block endogenous peroxidase activity. The slides were incubated at 4 °C overnight with primary antibody diluted with 3% BSA solution. The primary antibody information is as follows: PA28γ (PA5-21789, Invitrogen, Carlsbad, USA), 1:200; MEK1 (ab32091, Abcam, Cambridge, UK), 1:200; p-MEK1 (ab96379, Abcam, Cambridge, UK), 1:200; and Ki67 (ab66155, Abcam, Cambridge, UK), 1:100. Then, tissue sections were incubated with the matching secondary antibodies (Zsbio, Beijing, CHN) for 30 min at 37 °C and visualized with 2,3-diaminobenzidine (DAB) hydrochloride. Images of the tissue sections were acquired with an Aperio Scanscope (Aperio, USA). IHC scores were based on the staining intensity and density of positive cells, which were assessed and confirmed by two experienced pathologists independently.

### Masson’s trichrome staining

Masson’s trichrome staining uses a classic three-color method and was performed following the protocols of a previous report. Tissue paraffin sections were deparaffinized, rehydrated in an alcohol gradient, rinsed, and then processed with a Masson’s Trichrome Stain Kit (D026-1-1, NJJCBIO, Nanjing, CHN). Sections were scanned by an Aperio Scanscope (Aperio, USA) after treatment with nuclear staining solution, cytoplasmic staining solution, and counterstaining solution. Collagen fibers are shown in blue, cytoplasm and muscle tissue in red, and nuclei in blue–purple images. The collagen volume fraction area was read by ImageJ software.

### Data acquisition and bioinformatic analysis

The gene expression of various types of fibrosis was downloaded from GeneCards (https://www.genecards.org). The screening conditions and results were set as follows: keywords: oral submucosal fibrosis, 173 related genes; keywords: pulmonary fibrosis, 2349 related genes; keywords: liver fibrosis, 1778 related genes; keywords: kidney fibrosis, 1792 related genes; keywords: myocardial fibrosis, 883 related genes (Table S2). Finally, the candidate genes were found by combining the prognostic genes from TCGA, and the gene expression and clinical information data of OSCC were downloaded from TCGA (belonging to the head and neck squamous cell carcinoma subclass). The gene expression level was divided into high and low by the median. Then, the Kaplan–Meier method was used to draw survival curves, and the log‐rank test was conducted to determine the correlation between gene expression and the prognosis of OSCC patients. The correlation analysis of genes was obtained through the online website tool Timer2.0 (http://timer.cistrome.org/).

### Cell culture and reagent treatment

Two epithelial cell lines, HOK16E6E7 (HOK) and HaCat, were used in this research. The human immortalized oral keratinocyte cell line HOK was provided by Dr. Xuan Liu (Charles R. Drew University of Medicine and Science) and was cultured in keratinocyte serum-free medium (KSFM; Gibco BRL Life Technology Grand Island, NY, USA) supplemented with epidermal growth factor. The HaCat cell line was provided by Xiangya Stomatological Hospital & School of Stomatology. All cell lines were identified by short tandem repeat (STR) analysis and the amelogenin gene before use. The HaCat cell lines and 293T cells were cultured in DMEM (Gibco, Grand Island, NY, USA) supplemented with 10% fetal bovine serum (HyClone, UT, USA). All cells were cultured in T75 tissue culture flasks in a humidified incubator at 37 °C with 5% CO_2_. Plasmid transfection using Lipofectamine® 2000 was carried out following the manufacturer’s instructions. Cycloheximide (CHX, Sigma–Aldrich, MO, USA) and MG132 (M7449, Sigma-Aldrich, MO, USA) were also used in the cell experiments.

### Plasmid and siRNA transfection

PCR-amplified human PA28γ and BRAF were cloned into pcDNA3.1/hygro (+)-Flag or Myc vectors to produce a Flag-tagged Flag-PA28γ construct or myc-tagged myc-BRAF construct. The HA-MEK1 overexpression plasmid pLVX-ORF-HA-MEK1 was purchased from Sino Biological (#HG10661-CY, Beijing, CHN). pRK5-HA-Ubiquitin-K63 (#17606), pRK5-HA-Ubiquitin-K48 (#17605), and pRK5-HA-Ubiquitin-WT (#17608) were gifts from Ted Dawson (Addgene). Transfection was performed with Lipofectamine RNAiMAX Transfection Reagent (Invitrogen, USA) following the manufacturer’s protocol. The target sequences of PA28γ knockdown were as follows: 5′‐GACAGAGATTGATGAGAAA‐3′ and 5′‐GGAAACAGTTGCAGAGCTA‐3′ for sh‐PA28γ1 and sh‐PA28γ2, respectively. The target sequences of MEK knockdown were as follows: siMEK1-1#: 5′-GAGGTTCTCTGGATCAAGT-3′; siMEK1-2#: 5′-GAGAGCAGATTTGAAGCAA-3′; siMEK1-3#: 5′-CCAGTGGAGTGTTCAGTCT-3′. siMEK1–3# was used in downstream experiments. The target sequences of BRAF knockdown were as follows: siBRAF-1#: GGAGTTACAGTCCGAGACA; siBRAF-2#: GCATCAATGGATACCGTTA; siBRAF-3: CATTTGGAACAGTCTACAA. Since the single siRNA could not achieve a satisfactory inhibiting effect, we mixed all three siRNAs to knockdown BRAF expression, and the downstream experiments were all performed under this condition. The siRNA-NC (siRNA-nontarget) was synthesized by RiboBio Co., Ltd, Guangzhou.

### Stable cell line generation

The pCMV-dR8.9-PA28γ recombinant lentivirus or pCMV-dR8.9 vector lentivirus (OE-PA28γ or OE-vector) was purchased from Sangon Biotech (Shanghai Sangon Co., Ltd.). HOK and HaCat cells were infected with OE-PA28γ or OE-vector. Selective culture medium containing puromycin was used to select the cells with stable expression of Flag-PA28γ or vector controls. The expression of Flag-PA28γ was detected by Q-PCR and western blot analysis.

### Real-time quantitative PCR

Total RNA was extracted from each sample using TRIzol reagent (Invitrogen, Grand Island, NY, USA) and reverse transcribed by using a PrimeScript RT reagent kit (Takara) following the manufacturer’s protocol. The primers used for Q-PCR were as follows: PA28γ forward: 5′-AAGGTTGATTCTTTCAGGGAGC-3′, PA28γ reverse: 5′-AGTGGATCTGAGTTAGGTCATGG-3′; MEK1 forward: 5′-CAATGGCGGTGTGGTGTTC-3′, MEK1 reverse: 5′-CCCACGGGAGTTGACTAGGAT-3′; BRAF forward: AGTACTCAGGAAAACACGACAT; BRAF reverse: CTTGGCGTGTAAGTAATCCATG; GAPDH forward: 5′- ACAACTTTGGTATCGTGGAAGG-3′, GAPDH reverse: 5′- GCCATCACGCCACAGTTTC -3′. Quantitative PCR was performed using the SYBR Green system (Takara, Kusatsu, Japan) and run in an ABI 7300 Real-time PCR instrument.

### Cell proliferation assay

A Cell Counting Kit-8 (Dojindo, Japan) was used to determine cell proliferation after treatment with arecoline. Epithelial cell suspension (200 μL, 2000 cells per well) was inoculated in a 96-well plate. The cells adhered to the plate and were starved overnight with serum-free medium. Then, the epithelial cells were treated with a concentration gradient of arecoline for 24, 48, and 72 h in a humidified incubator at 37 °C with 5% CO_2_. Then, 20 μL of CCK-8 solution was added to each well of the plate and incubated for 2 h. Finally, the OD was measured at 450 nm by a microplate reader.

### Western blot assay

The indicated cells were washed three times in 1× PBS and then lysed on ice in lysis buffer containing protease inhibitor cocktail. The extracted protein lysate samples were quantitatively analyzed by a BCA Protein Assay Kit (23225, Thermo Fisher, MA, USA) and mixed with loading buffer for denaturation (100 °C, 5 min). Protein samples of equal volume and mass were subjected to 8–15% SDS–PAGE and transferred to PVDF membranes (Millipore). Then, the membranes were incubated in 5% skimmed milk at room temperature for 1 h and incubated overnight at 4 °C with primary antibodies. Finally, the protein bands incubated in ECL were visualized by chemiluminescence.

The primary antibodies used for western blot analysis are listed below. PA28γ (PA5-21789, Invitrogen, Carlsbad, USA), 1:2000. BRAF (ab33899, Abcam, Cambridge, UK), 1:2000. MEK1 (ab32091, Abcam, Cambridge, UK), 1:2000. p-MEK1 (ab96379, Abcam, Cambridge, UK), 1:2000. ERK1/2 (ab17942, Abcam, Cambridge, UK), 1:2000. p-ERK1/2 (ab201015, Abcam, Cambridge, UK), 1:2000. Vimentin (ab92547, Abcam, Cambridge, UK), 1:2000. E-Cadherin (14472, Cell Signaling Technology, MA, USA), 1:1000. Ubiquitin mAb (1107, PTM BIO, Hangzhou, CHN), 1:2000. Ubiquitin (PTM-1107, PTM BIO, Hangzhou, CHN), 1:2000. Myc-Tag (2276S, Cell Signaling Technology, MA, USA), 1:1000. Flag-Tag (F1804, Sigma–Aldrich, MO, USA), 1:1000. HA-Tag (AF0039, Beyotime, Shanghai, CHN), 1:1000. GAPDH (5174S, Cell Signaling Technology, MA, USA), 1:1000.

### Immunoprecipitation assay

For exogenous immunoprecipitation, cells were washed with cold PBS, harvested, and lysed in an IP lysis buffer (0.1% SDS, 1% Triton-HCl, 150 mM NaCl, 0.5 mM EDTA, 1 mM DTT) mixture of protease inhibitors for 30 min on ice. The cell lysate was centrifuged at 4 °C and 12000 rpm for 10 min, and then the supernatant was obtained. The protein concentration was measured by the BCA quantitative method to ensure that the protein quality of each sample was as close as possible to approximately 1000 μg. Approximately 20 μg of total protein was extracted as an input control. The lysates were incubated with 20 μL of anti-Flag tag affinity gel antibody (651502, Biolegend, CA, USA) overnight at 4 °C. The precipitated protein complex was harvested by centrifugation, and then the beads were washed with cold immunoprecipitation buffer three times. The precipitated agarose was boiled to release binding protein, suspended in SDS–PAGE together with loading buffer for electrophoresis, and transferred to PVDF membranes. and immunoblotted with primary antibodies. For endogenous immunoprecipitation, lysates were incubated with 4 μg of anti-PA28γ, anti-BRAF, or anti-MEK1 antibody overnight at 4 °C, and 20 μL of protein A + G agarose (P2019, Beyotime, Shanghai, CHN) was added and incubated for an additional 2 h. The electrophoresis, transfer and strip exposure analyses were the same as those used for western blotting.

### Immunofluorescence assay

Cells were seeded in glass coverslips in a 24-well plate, washed 3 times with PBS, and fixed with 4% paraformaldehyde for 30 min. The glass coverslips were blocked with 3% BSA in PBS for 1 h at room temperature and incubated with primary antibody at 4 °C overnight. The next day, the slides were washed 3 times with PBS and incubated with the fluorescent secondary antibody conjugated with fluorochrome for 60 min at 37 °C. The cell nuclei were stained with DAPI-containing ProLong Gold antifade reagent (P36935, Invitrogen, Carlsbad, USA). Finally, cell images were observed and captured with immunofluorescence or laser scanning confocal microscopy.

### Ubiquitination assay

Cells were transfected with the indicated plasmids for 48 h and lysed using denatured buffer (6 M guanidine-HCl [pH 8.0], 0.1 M Na2HPO4/NaH2PO4, and 10 mM imidazole) containing 5 mM N-ethylmaleimide to prevent deubiquitylation. The cell lysates were immunoprecipitated using the indicated antibodies, washed, and subjected to immunoblotting analysis. The detailed procedure of the ubiquitination assay was described previously [49].

### Cell migration assay

Epithelial cells treated with arecoline (20 μg/mL) for 30 days or stable PA28γ-overexpressing epithelial cells (2 × 10^5^ per well) were seeded into the upper compartment of a 24-well transwell chamber with serum-free DMEM. The lower compartment contained DMEM supplemented with 10% FBS. The migrated cells were fixed with 4% paraformaldehyde and stained with crystal violet staining solution. The membrane of the Transwell chamber was fixed with a resin sealant and photographed with an Aperio Scanscope (Aperio, USA). Three replicates were obtained for each group.

### Wound healing assay

The indicated cells were reseeded onto 6 cm dishes to create an 80–90% confluent monolayer. The cell monolayer was scraped in a straight line with a 200 μL pipette tip to create a “scratch”. The first image of the scratch was acquired (0 h), and cells were cultured in the incubator at 37 °C for 24 and 48 h to acquire the image. The wound healing percentage (%) = migrated cell surface area/total surface area 100. The gray value of the scratch area was read by ImageJ software.

### OSF mouse model

Twenty-four pathogen-free female mice (BALB/c, six weeks old) were purchased from the State Key Laboratory of Oral Diseases, Sichuan University. After two weeks of normal feeding in the animal laboratory of the West China Hospital of Stomatology, the mice were equally divided into four groups. The three groups of mice were treated with saline (PBS, 1 mg/ml), arecoline (ARE, Sigma-Aldrich, 31593, 1 mg/ml), and bleomycin (BLE, 1 mg/ml). Every 2 days, we used a 29-gauge insulin syringe (Ultra-Fine, BD) to administer 50 μl of drug treatment to the bilateral buccal mucosa of the mouse for 14 weeks. After the mice were sacrificed, the obtained buccal mucosa of the oral cavity was fixed (4% paraformaldehyde), dehydrated, and embedded for subsequent experiments.

### Data collection and statistical analysis

The IHC score scale was defined according to previous studies: staining intensities (scale, 1–4) and the density of positively stained cells (scale, 1–4) were determined, and final score: staining intensity multiplied by staining proportion. All experiments in this manuscript were repeated at least three times. Statistical analysis was performed using GraphPad Prism 8.0 software. Statistical significance was assessed by two-tailed Student’s t test or two-way ANOVA, with a value of *P* < 0.05 indicating statistical significance. Student’s t test was used to determine the significance of the difference between the two groups, and analysis of variance (ANOVA) was used to compare more than two groups. Pearson’s correlation coefficient was used for correlation analysis. For all tests, a *p* value less than 0.05 was considered significantly different (*p < 0.05, **p < 0.01, ***p < 0.001, ****p < 0.0001).

## Supplementary information

reproducibility checklist

Supplementary Table S1

Supplementary Table S1

Supplementary Table S2

Supplementary Table S2

Supplementary Figure S1

Supplementary Figure S2

Supplementary Figure S3

Supplementary Figure S4

Supplementary Figure S5

Supplementary Figure S6

Supplementary Figure S7

## Data Availability

All data generated or analyzed during this study are included in this published article and its supplementary information files.
