# Supplementary figures and images for: Identification of a BRAF/PA28γ/MEK1 signaling axis and its role in epithelial-mesenchymal transition in oral submucous fibrosis
Source: Cell Death Dis. 2022 Aug 12;13(8):701. doi: 10.1038/s41419-022-05152-6 (PMC9374740; doi:10.1038/s41419-022-05152-6)

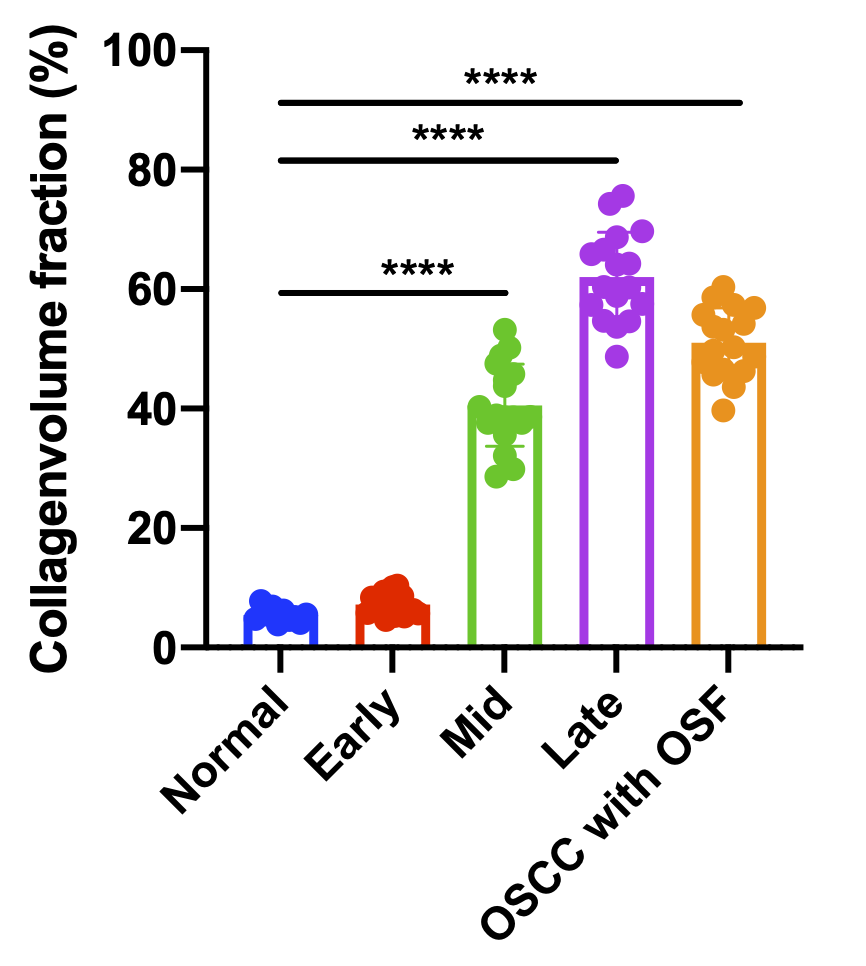

Supplement: Supplementary file 4 — Supplementary Table S2 [file 41419_2022_5152_MOESM4_ESM.tif]

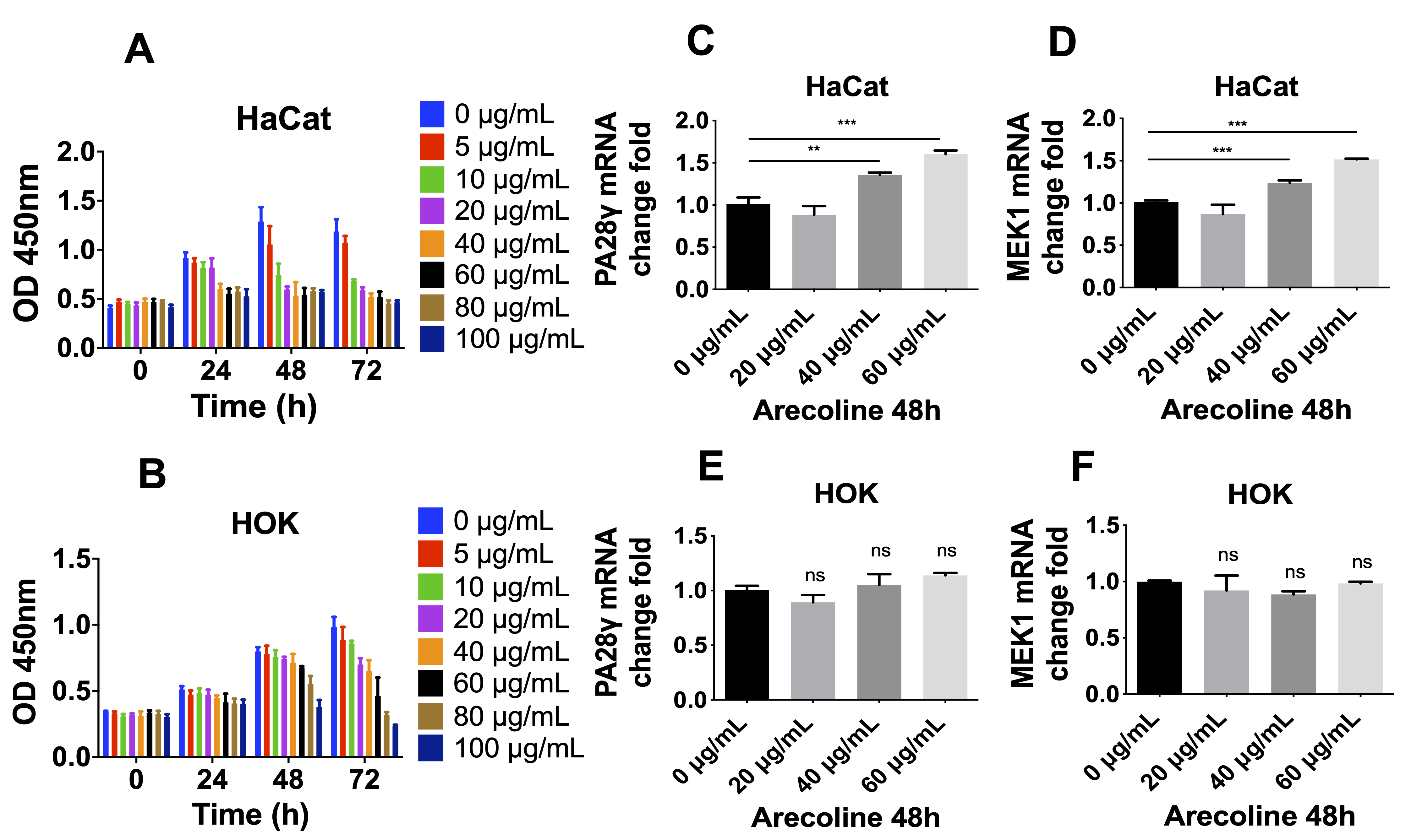

Supplement: Supplementary file 5 — Supplementary Table S2 [file 41419_2022_5152_MOESM5_ESM.tif]

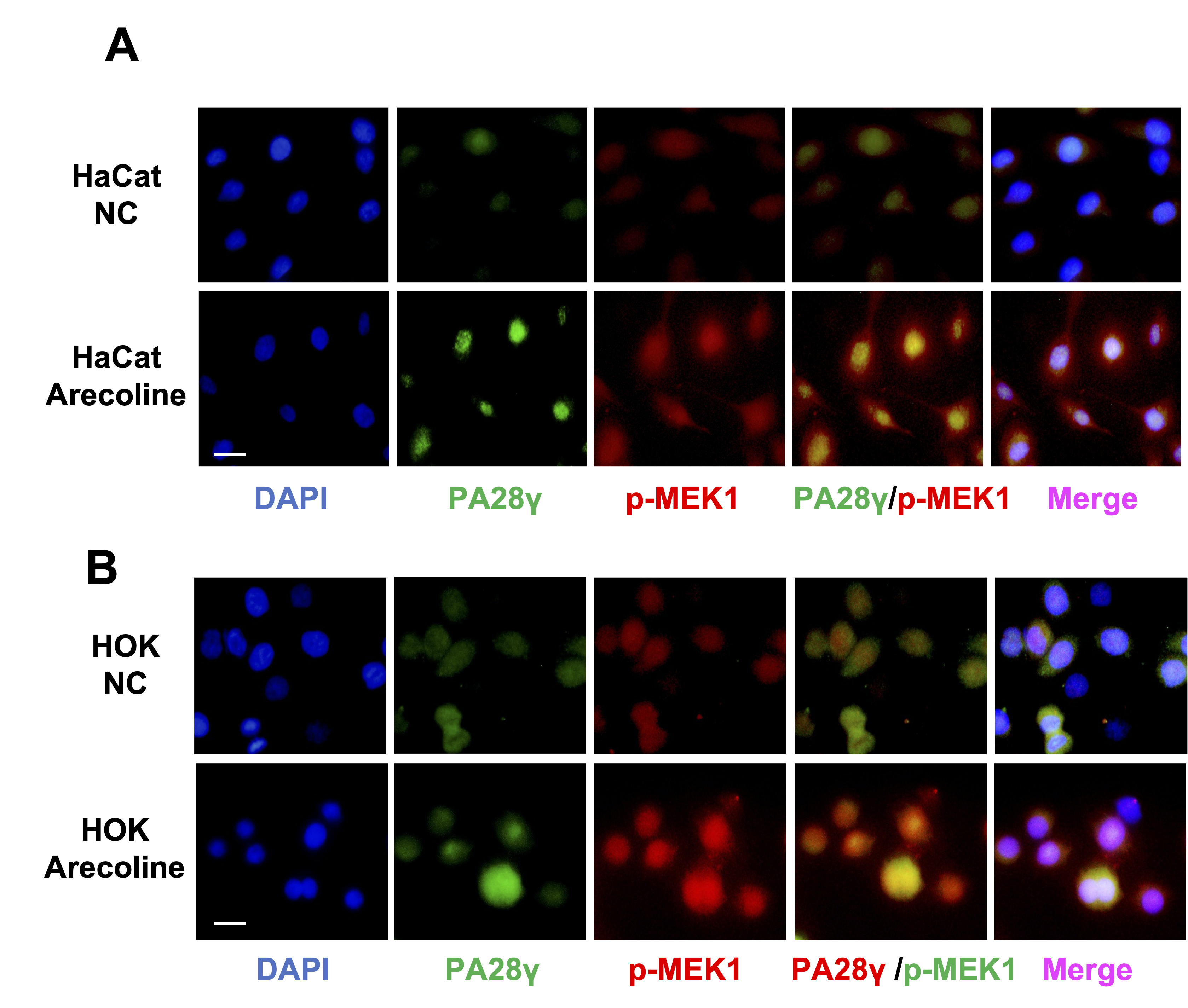

Supplement: Supplementary file 6 — Supplementary Figure S1 [file 41419_2022_5152_MOESM6_ESM.tif]

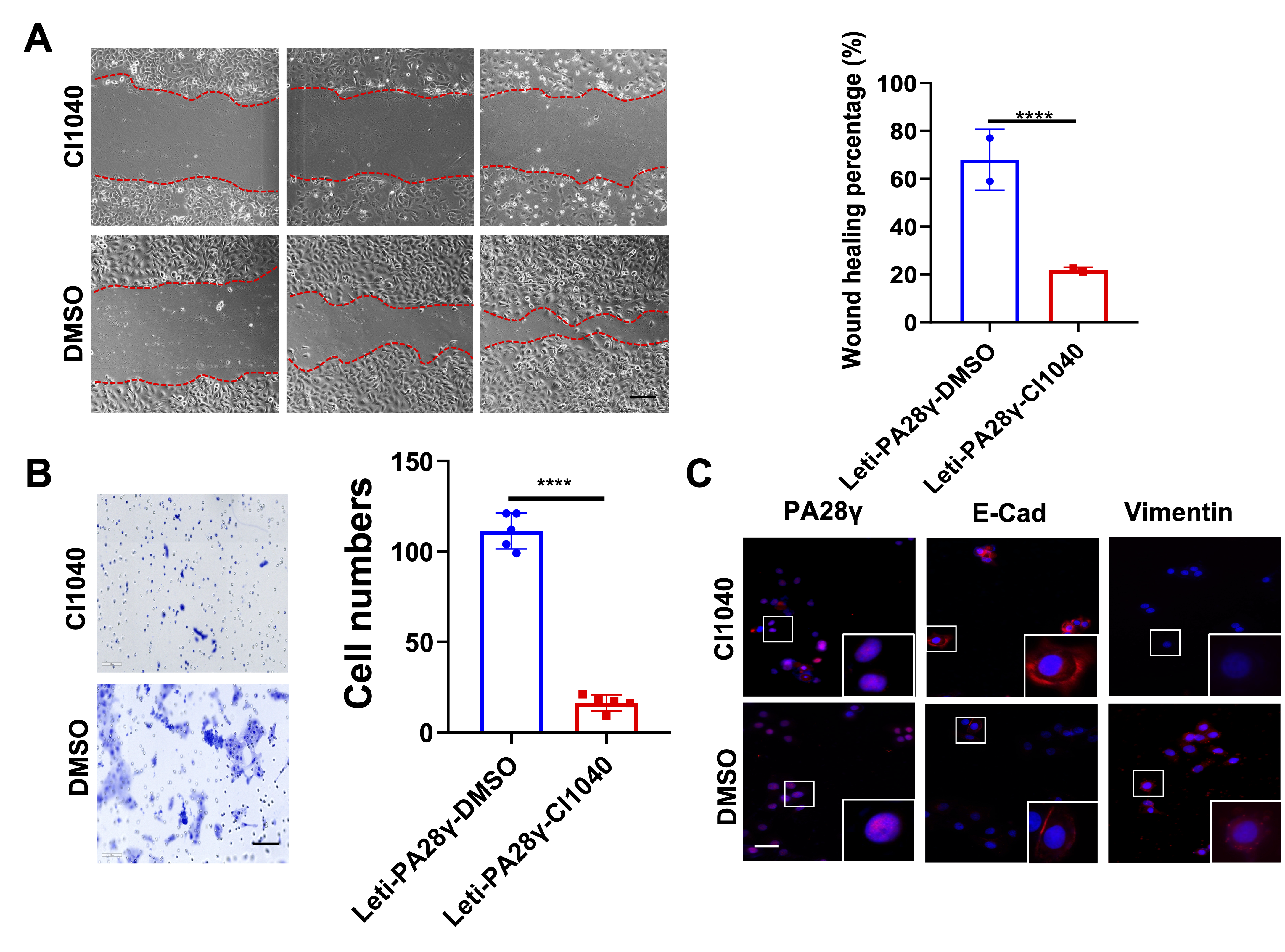

Supplement: Supplementary file 9 — Supplementary Figure S4 [file 41419_2022_5152_MOESM9_ESM.tif]

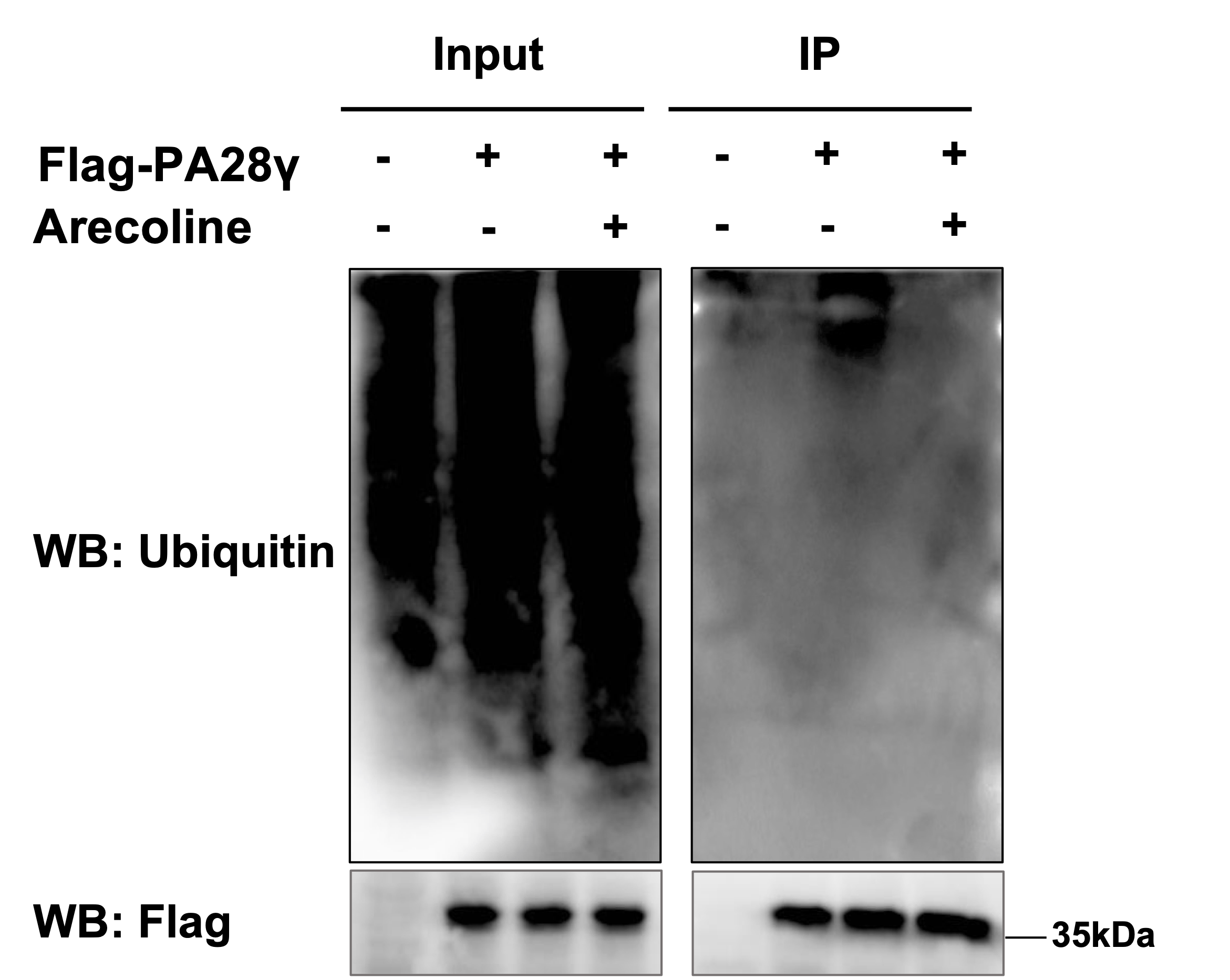

Supplement: Supplementary file 11 — Supplementary Figure S6 [file 41419_2022_5152_MOESM11_ESM.tif]
