# Supplementary material for: Identification of a BRAF/PA28γ/MEK1 signaling axis and its role in epithelial-mesenchymal transition in oral submucous fibrosis
Source: Cell Death Dis. 2022 Aug 12;13(8):701. doi: 10.1038/s41419-022-05152-6 (PMC9374740; doi:10.1038/s41419-022-05152-6)

Uncropped whole blots of figures used in this study

Corresponds to Fig.2A, first and second column.

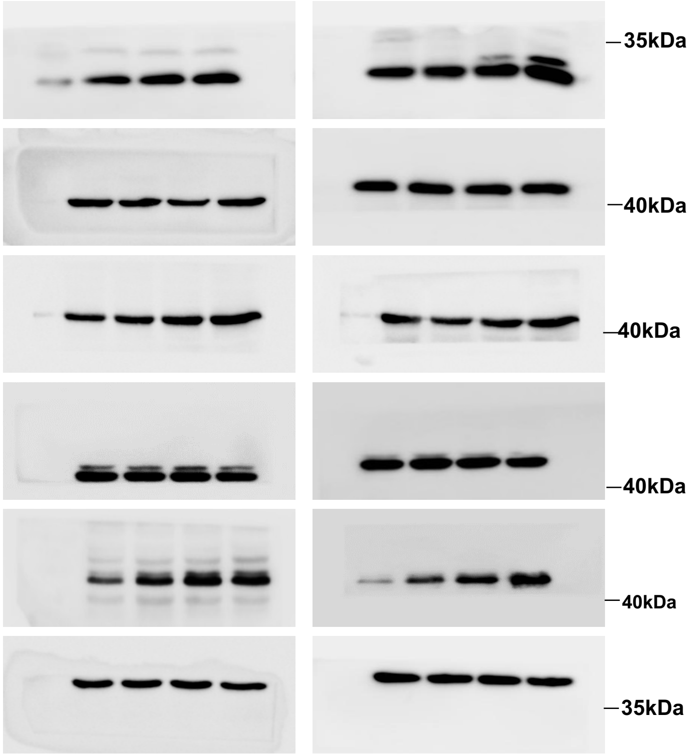

Corresponds to Fig. 2B, first and second column.

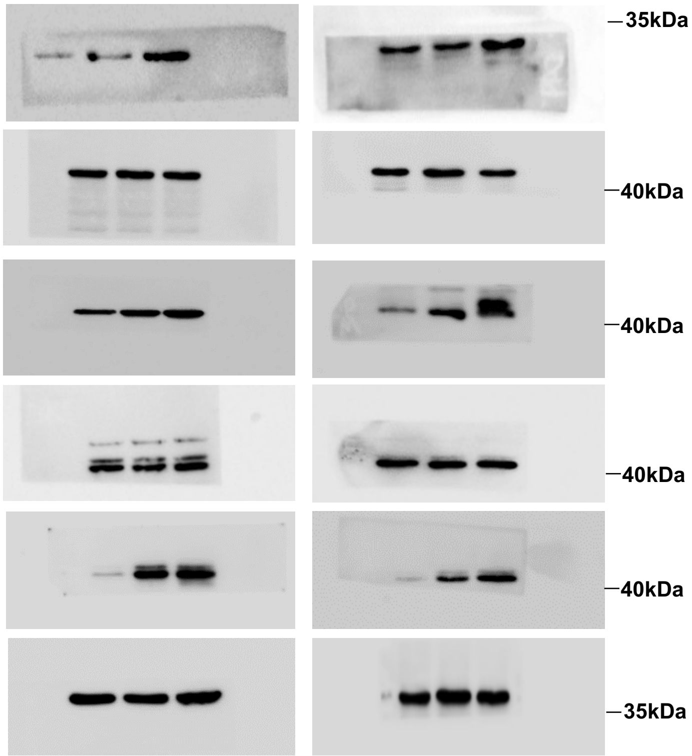

Corresponds to Fig. 2I, first and second column.

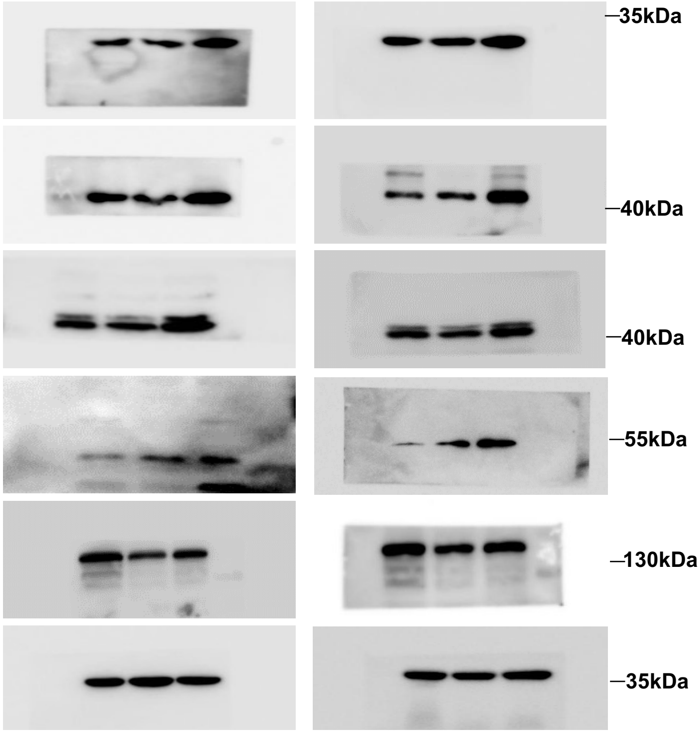

Uncropped whole blots of figures used in this study

Corresponds to Fig. 3A, first row and first column.

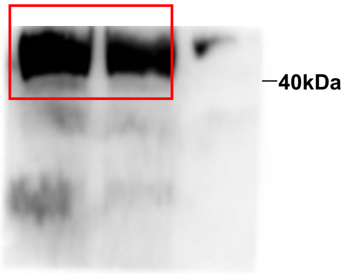

Corresponds to Fig. 3A, second row and first column.

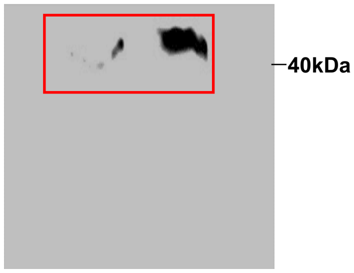

Corresponds to Fig. 3B, first row.

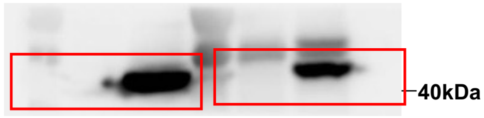

Corresponds to Fig. 3B, second row.

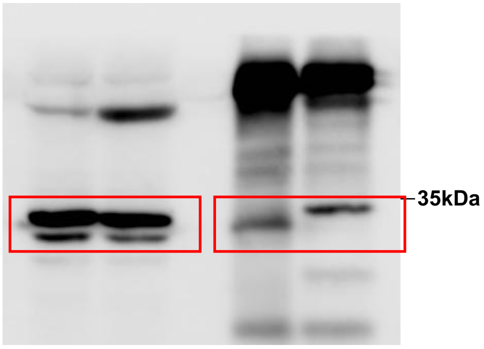

Corresponds to Fig. 3A, first row and second column.

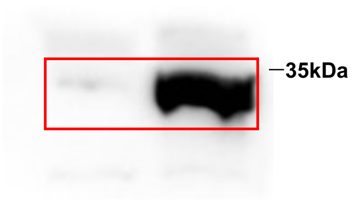

Corresponds to Fig. 3A, second row and second column.

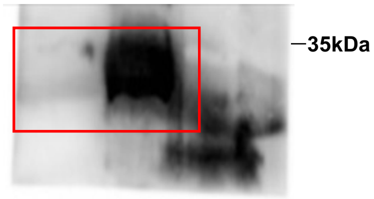

Corresponds to Fig. 3C, first row.

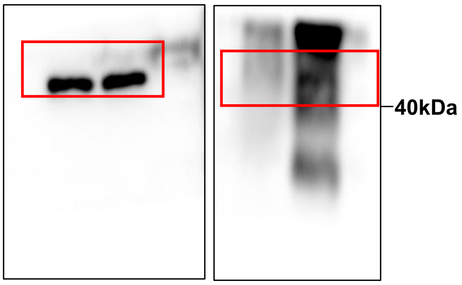

Corresponds to Fig. 3D, first row.

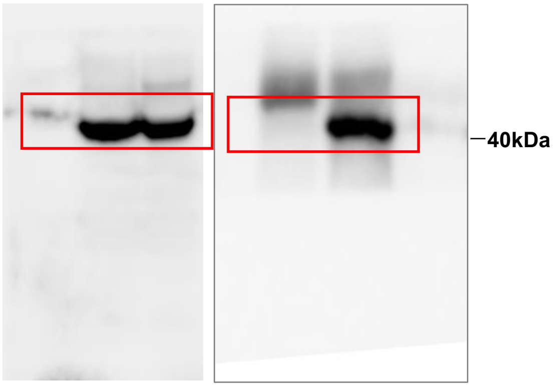

Corresponds to Fig. 3C, second row.

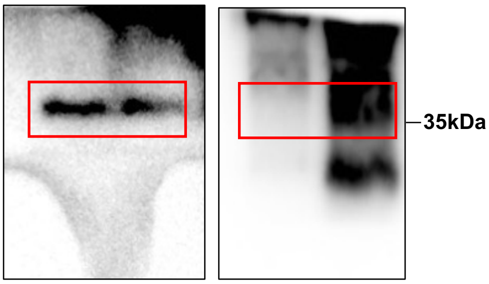

Corresponds to Fig. 3D, second row.

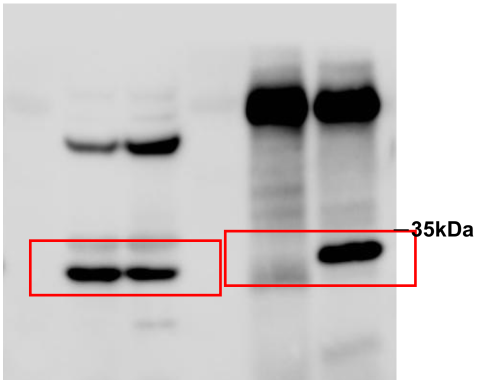

Corresponds to Fig. 3G, first row, first column.

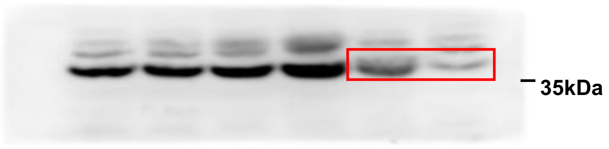

Corresponds to Fig. 3G, first row, second column.

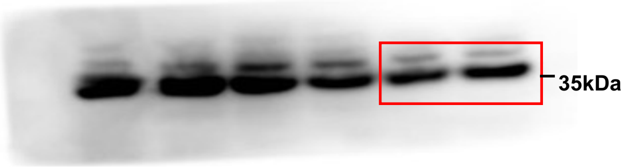

Corresponds to Fig. 3G, second row, first column.

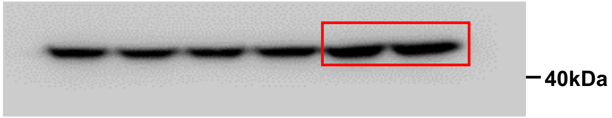

Corresponds to Fig. 3G, second row, second column.

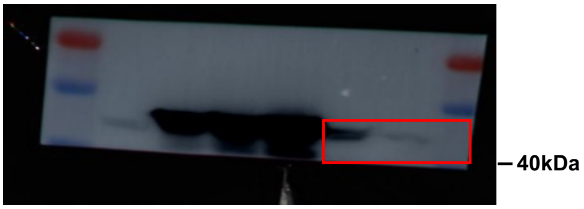

Corresponds to Fig. 3G, third row, first column.

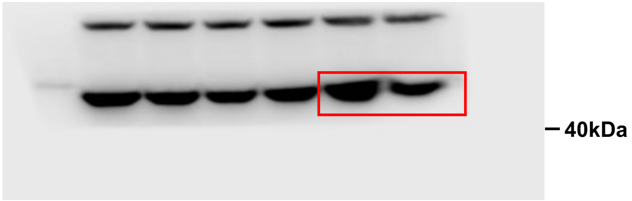

Corresponds to Fig. 3G, third row, second column.

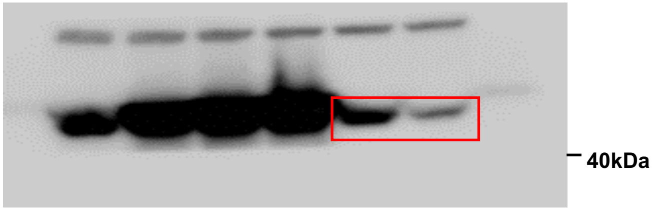

Corresponds to Fig. 3G, fourth row, first column.

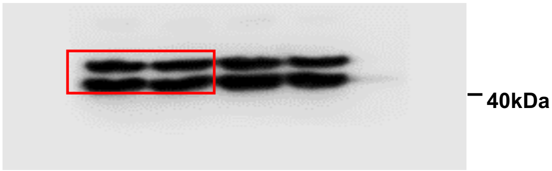

Corresponds to Fig. 3G, fourth row, second column.

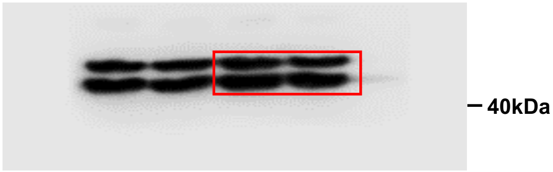

Corresponds to Fig. 3G, fifth row, first column.

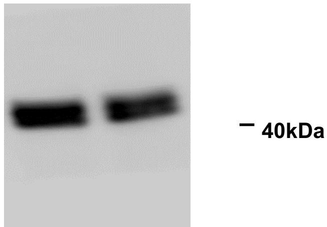

Corresponds to Fig. 3G, fifth row, second column.

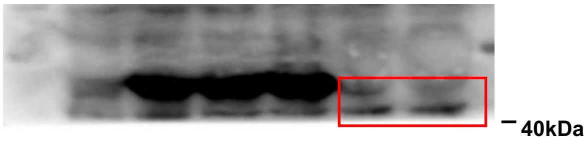

Corresponds to Fig. 3G, sixth row, first column.

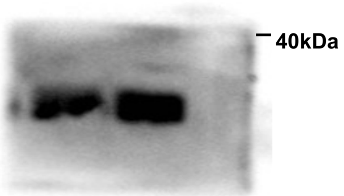

Corresponds to Fig. 3G, sixth row, second column.

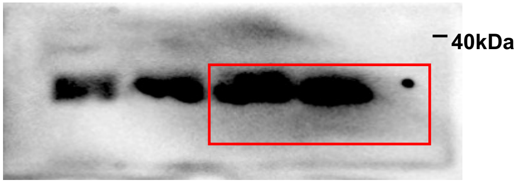

Corresponds to Fig. 3H, first row.

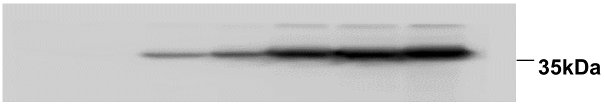

Corresponds to Fig. 3H, second row.

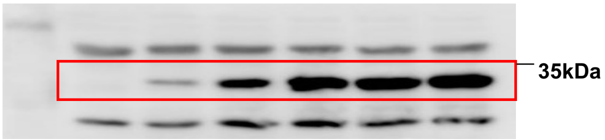

Corresponds to Fig. 3H, third row.

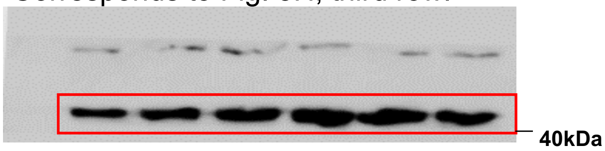

Corresponds to Fig. 3H, fourth row.

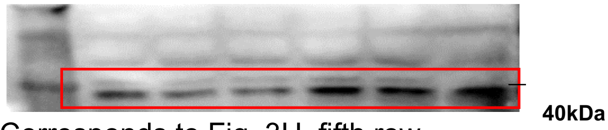

Corresponds to Fig. 3H, fifth row.

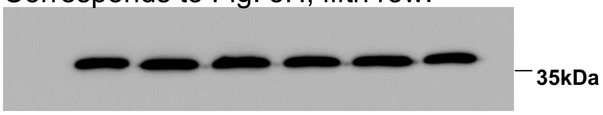

Corresponds to Fig. 4F, first row, first column

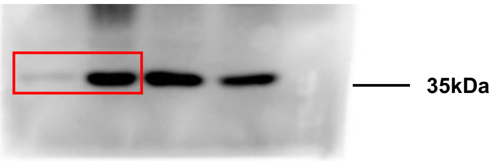

Corresponds to Fig. 4F, second row, first column

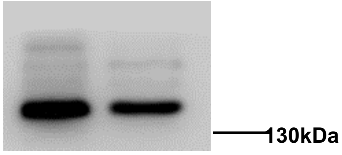

Corresponds to Fig. 4F, third row, first column

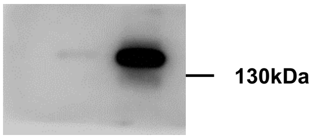

Corresponds to Fig. 4F, fourth row, first column

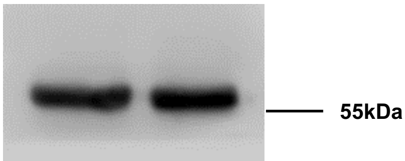

Corresponds to Fig. 4F, fifth row, first column

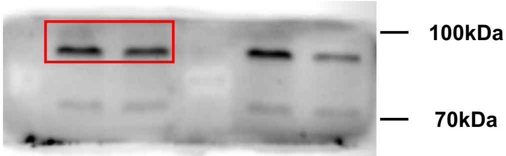

Corresponds to Fig. 4F, sixth row, first column

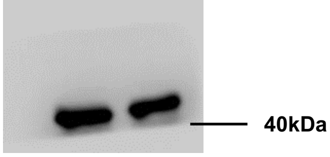

Corresponds to Fig. 4F, seventh row, first column

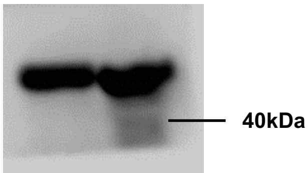

Corresponds to Fig. 4F, eighth row, first column

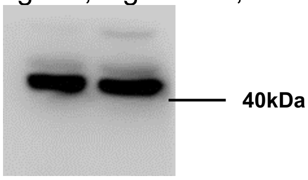

Corresponds to Fig. 4F, ninth row, first column

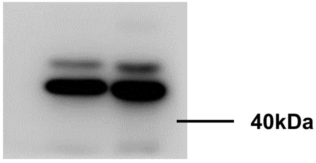

Corresponds to Fig. 4F, tenth row, first column

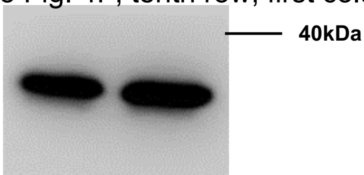

Corresponds to Fig. 4F, first row, second column

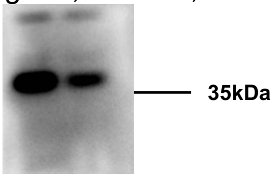

Corresponds to Fig. 4F, second row, second column

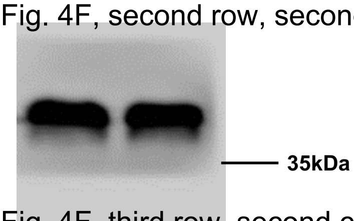

Corresponds to Fig. 4F, third row, second column

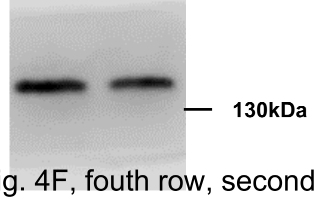

Corresponds to Fig. 4F, fouth row, second column

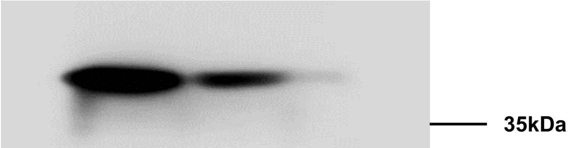

Corresponds to Fig. 4F, fifth row, second column

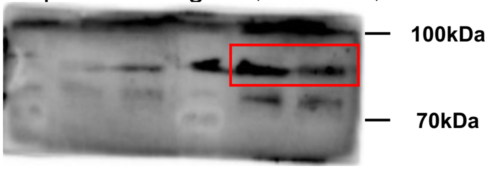

Corresponds to Fig. 4F, sixth row, second column

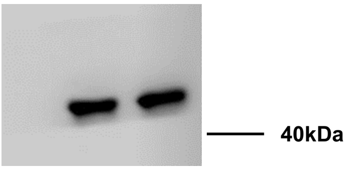

Corresponds to Fig. 4F, seventh row, second column

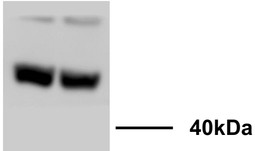

Corresponds to Fig. 4F, eighth row, second column

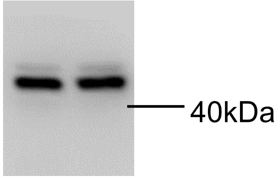

Corresponds to Fig. 4F, ninth row, second column

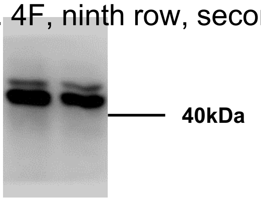

Corresponds to Fig. 4F, tenth row, second column

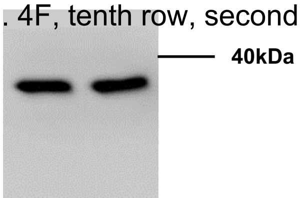

Uncropped whole blots of figures used in this study

Corresponds to Fig. 5A, first row and first column.

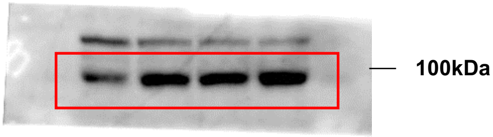

Corresponds to Fig. 5A, first row and second column.

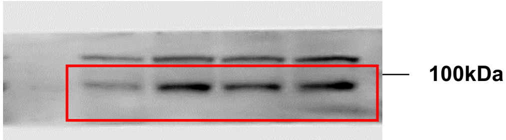

Corresponds to Fig. 5A, second row and first column.

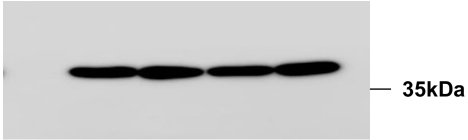

Corresponds to Fig. 5A, second row and second column.

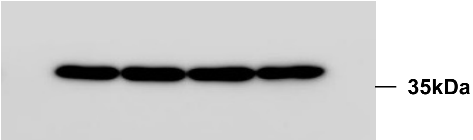

Corresponds to Fig. 5B, first row and first column.

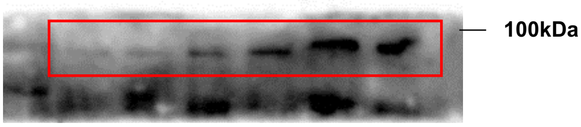

Corresponds to Fig. 5B, first row and second column.

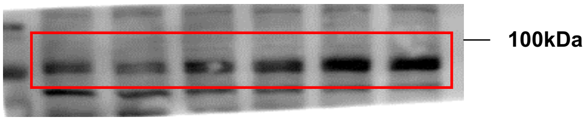

Corresponds to Fig. 5B, second row and first column.

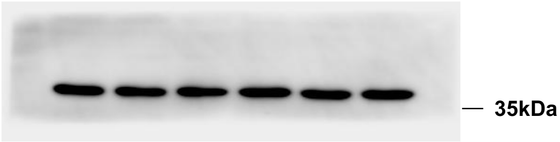

Corresponds to Fig. 5B, second row and second column.

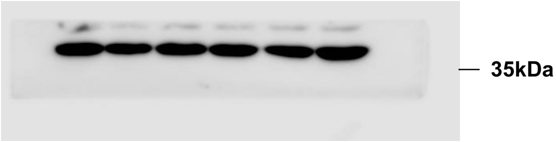

Corresponds to Fig. 5C, first row and first column.

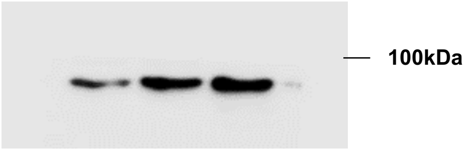

Corresponds to Fig. 5C, first row and second column.

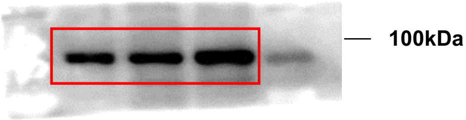

Corresponds to Fig. 5D, first row.

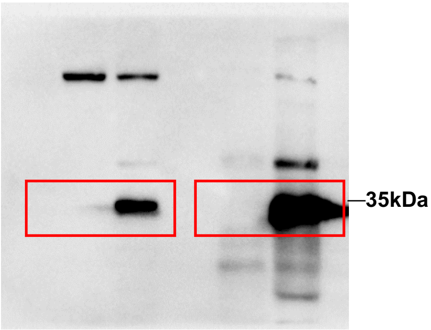

Corresponds to Fig. 5C, second row and first column.

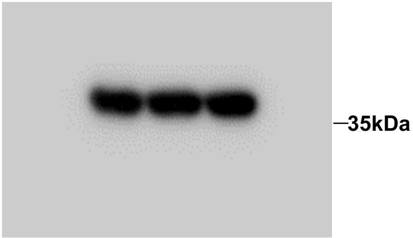

Corresponds to Fig. 5C, second row and second column.

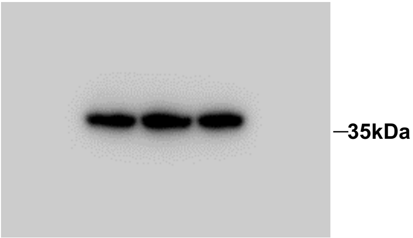

Corresponds to Fig. 5D, second row.

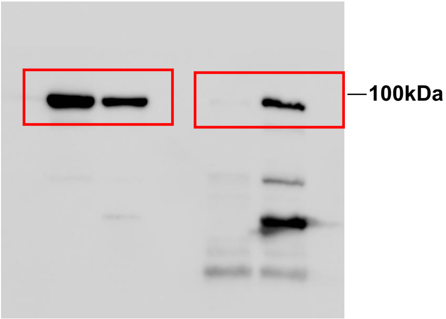

Corresponds to Fig. 5E, first row.

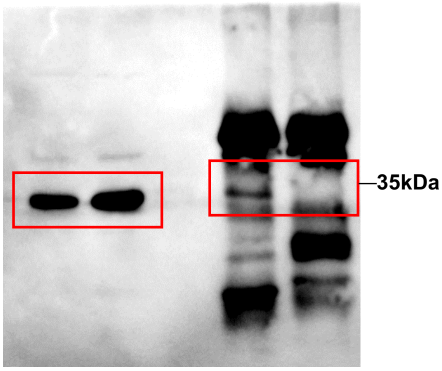

Corresponds to Fig. 5E, second row.

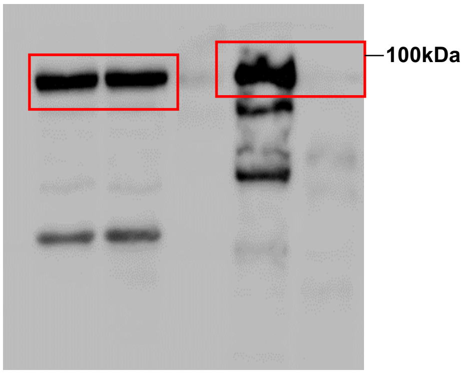

Uncropped whole blots of figures used in this study

Corresponds to Fig. 5F, first row.

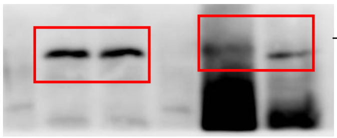

Corresponds to Fig. 5F, second row.

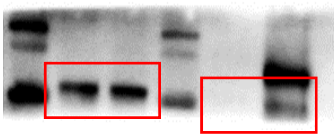

Corresponds to Fig. 5G, first row.

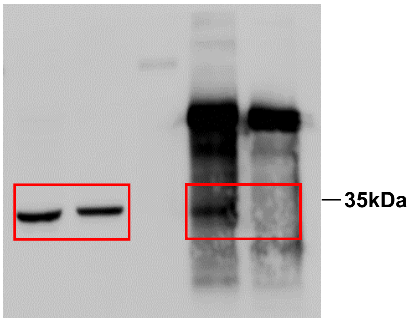

Corresponds to Fig. 5G, second row.

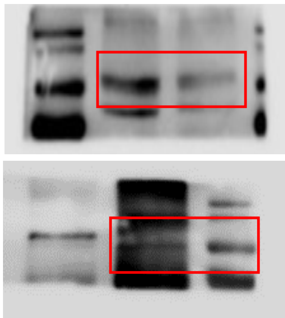

Corresponds to Fig. 5G, first row, first column.

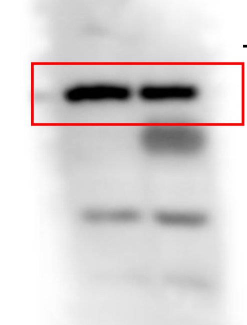

Corresponds to Fig. 5G, first row, second column.

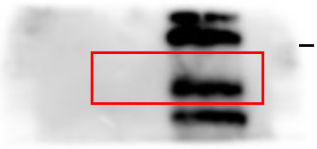

Corresponds to Fig. 5L, first row.

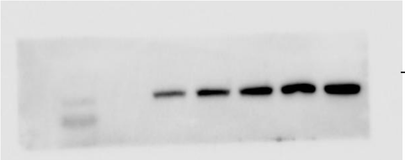

Corresponds to Fig. 5L, second row.

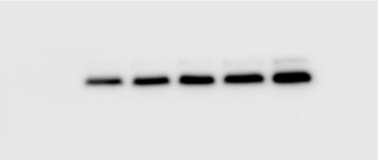

Corresponds to Fig. 5L, third row.

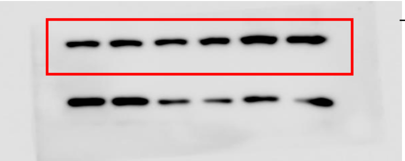

Corresponds to Fig. 5L, fourth row.

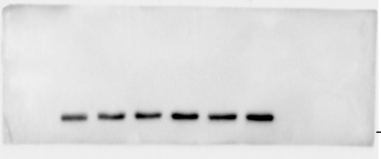

Corresponds to Fig. 5L, fifth row.

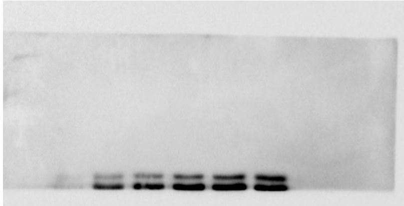

Corresponds to Fig. 5L, sixth row.

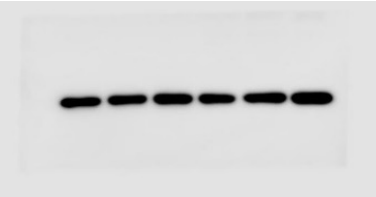

Corresponds to Fig. 5I, second row, first column.

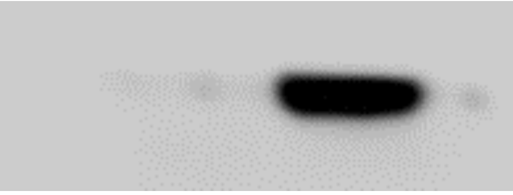

Corresponds to Fig. 5I, second row, second column.

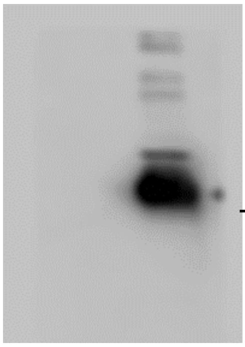

Corresponds to Fig. 5I, third row, first column.

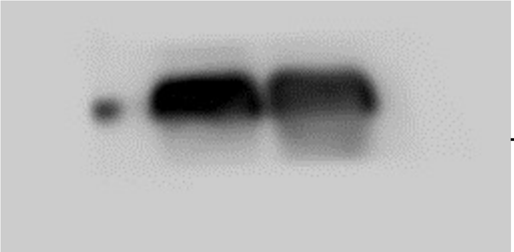

Corresponds to Fig. 5I, third row, second column.

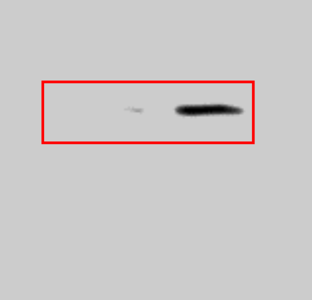

Uncropped whole blots of figures used in this study

Corresponds to Fig. 5J first row, first column.

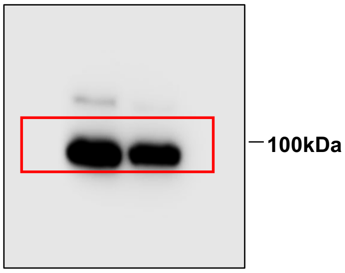

Corresponds to Fig. 5J second row, first column.

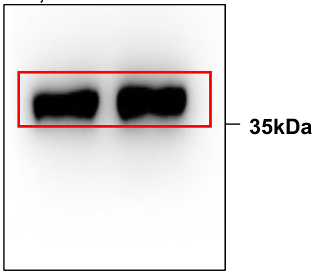

Corresponds to Fig. 5J third row, first column.

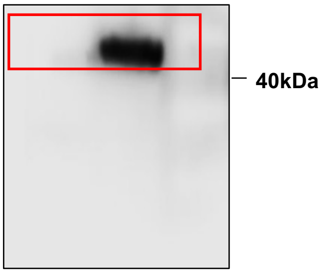

Corresponds to Fig. 5J first row, second column.

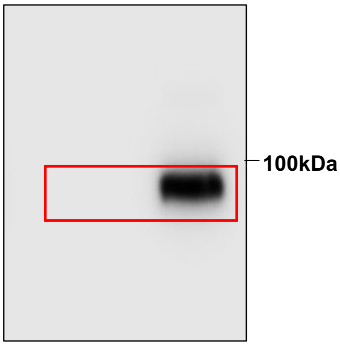

Corresponds to Fig. 5J second row, second column.

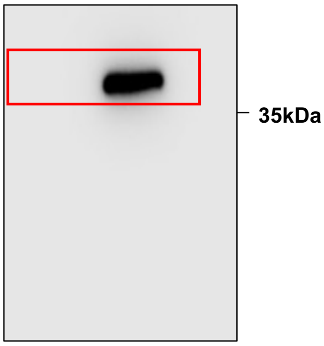

Corresponds to Fig. 5J third row, second column.

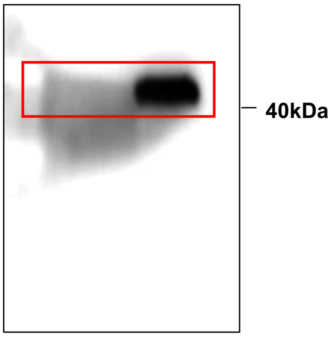

Corresponds to Fig. 5K first row, first column.

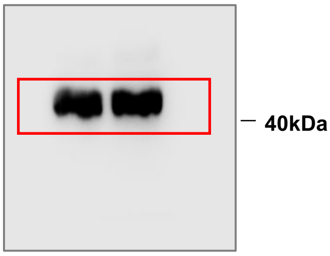

Corresponds to Fig. 5K second row, first column.

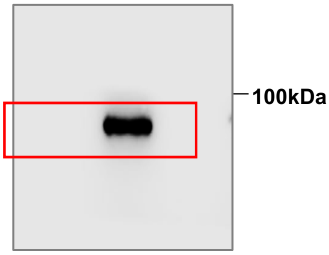

Corresponds to Fig. 5K third row, first column.

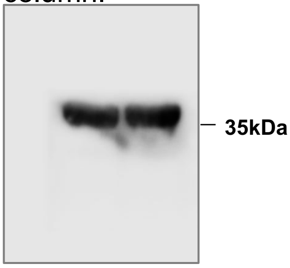

Corresponds to Fig. 5K first row, second column.

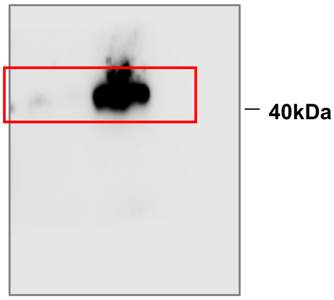

Corresponds to Fig. 5K second row, second column.

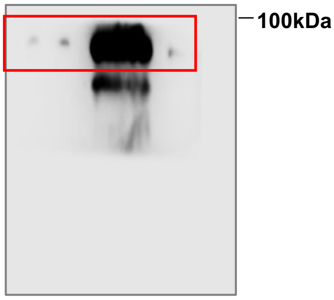

Corresponds to Fig. 5K third row, second column.

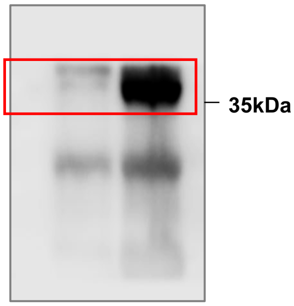

Corresponds to Fig. 5M, first row, first column.

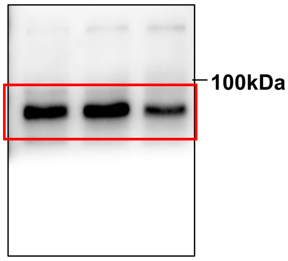

Corresponds to Fig. 5M, second row, first column.

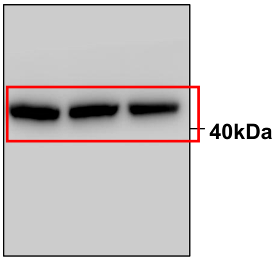

Corresponds to Fig. 5M, third row, first column.

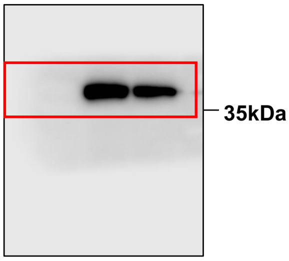

Corresponds to Fig. 5M, forth row, first column.

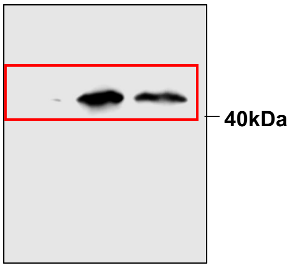

Corresponds to Fig. 5M, fifth row, first column.

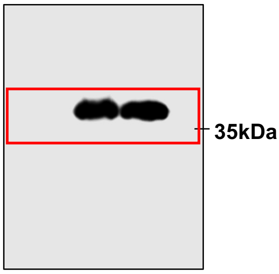

Corresponds to Fig. 5N, first row, first column.

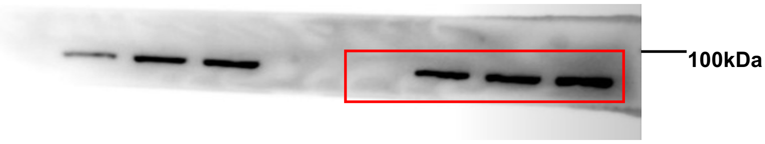

Corresponds to Fig. 5N, second row, first column.

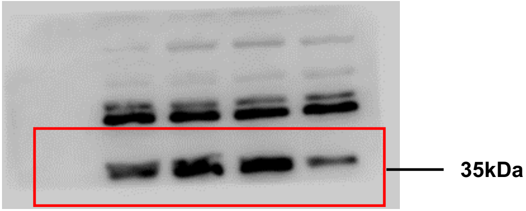

Corresponds to Fig. 5N, third row, first column.

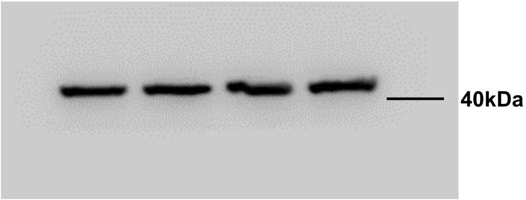

Corresponds to Fig. 5N, fourth row, first column.

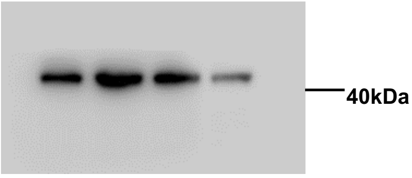

Corresponds to Fig. 5K, fifth row, first column.

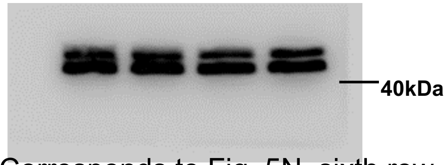

Corresponds to Fig. 5N, sixth row, first column.

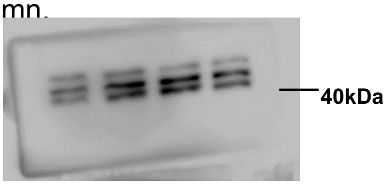

Corresponds to Fig 5N, seventh row, first column.

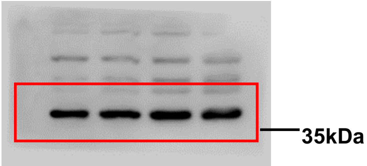

Corresponds to Fig. 6A, first row.

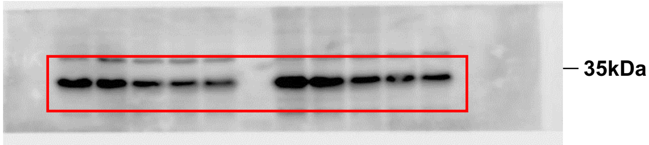

Corresponds to Fig. 6A, second row.

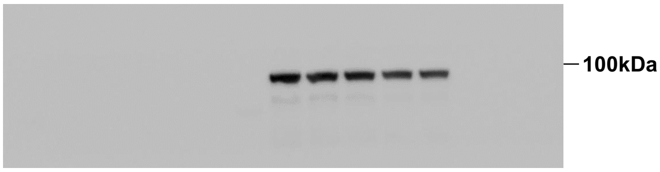

Corresponds to Fig. 6A, third row.

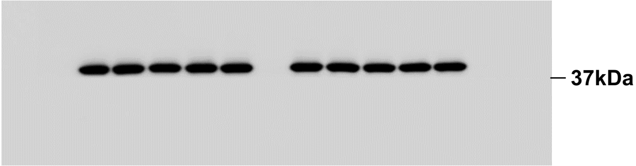

Corresponds to Fig. 6B, first row.

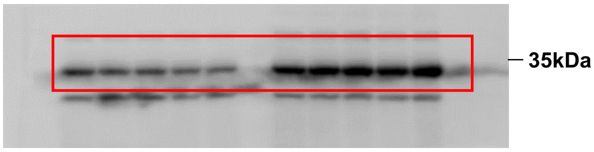

Corresponds to Fig. 6B, second row.

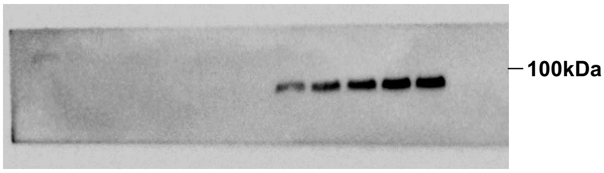

Corresponds to Fig. 6B, third row.

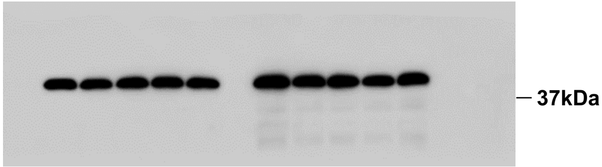

Corresponds to Fig. 6C, first row and first column.

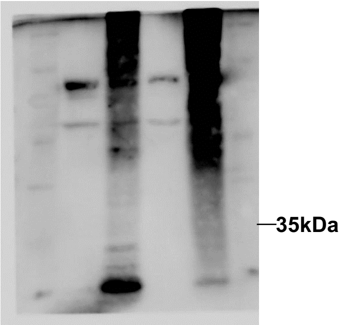

Corresponds to Fig. 6C, first row and second column.

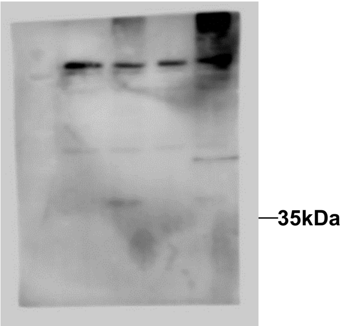

Corresponds to Fig. 6C, second row and first column.

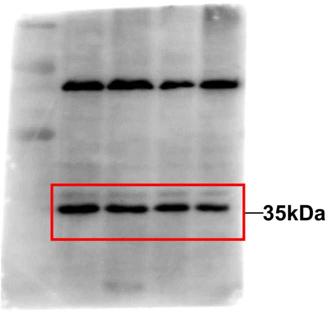

Uncropped whole blots of figures used in this study

Corresponds to Fig. 6C, second row and second column.

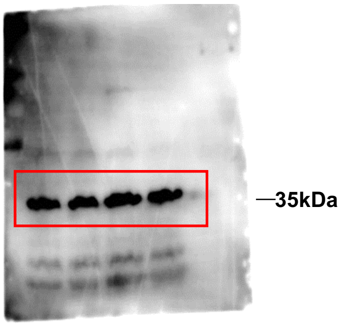

Corresponds to Fig. 6D, first row.

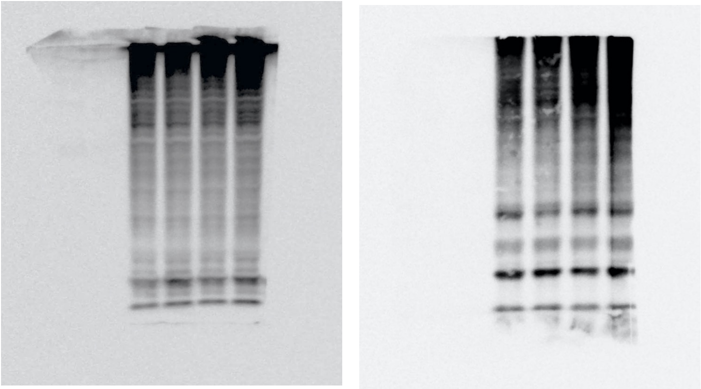

Corresponds to Fig. 6D, second row and first column.

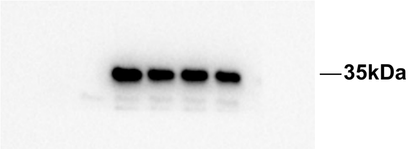

Corresponds to Fig. 6D, second row and second column.

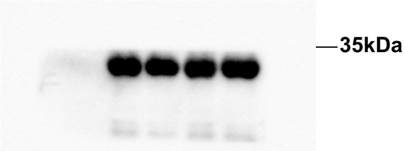

Corresponds to Fig. 6D, third row and first column.

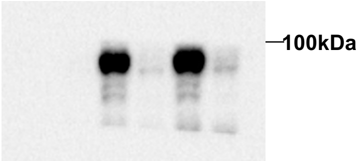

Corresponds to Fig. 6D, second row and first column.

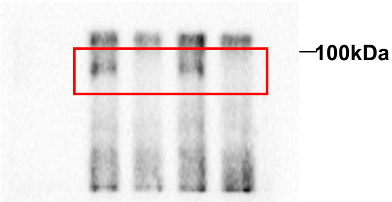

Corresponds to Fig. 6E, first row and first column.

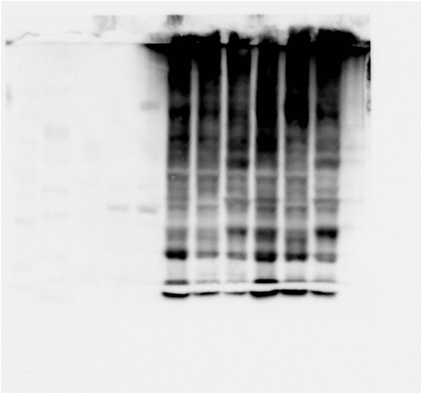

Corresponds to Fig. 6E, first row and second column.

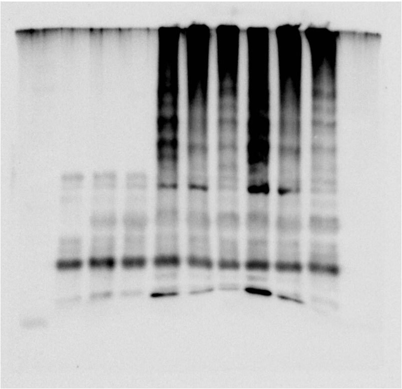

Corresponds to Fig. 6E, second row and first column.

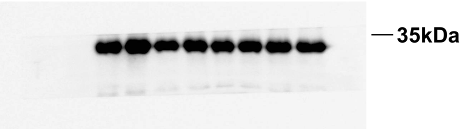

Corresponds to Fig. 6E, second row and second column.

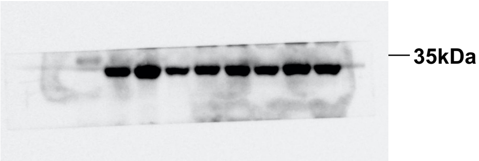

Corresponds to Fig. 6E, third row and first column.

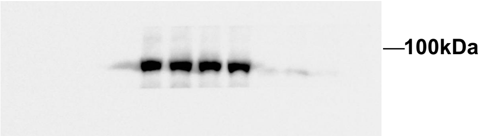

Corresponds to Fig. 6E, third row and third column.

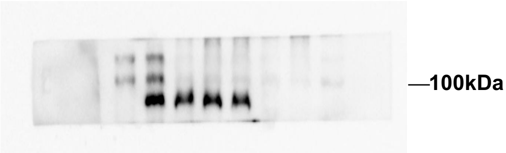

Uncropped whole blots of figures used in this study

Corresponds to Fig. S3A, first row.

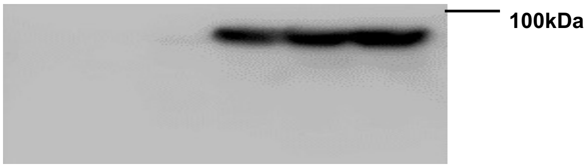

Corresponds to Fig. S3A, second row.

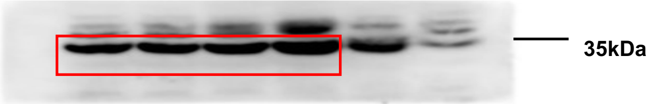

Corresponds to Fig S3A, third row.

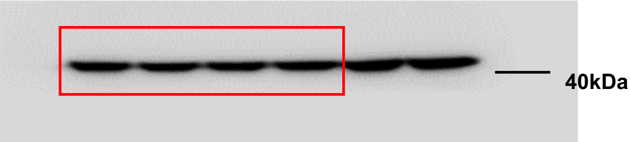

Corresponds to Fig. S3A, fourth row.

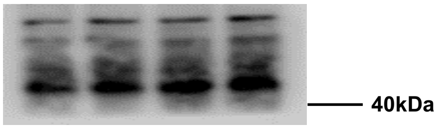

Corresponds to Fig. S3A, fifth row.

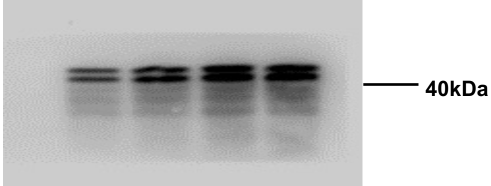

Corresponds to Fig. S3A, sixth row.

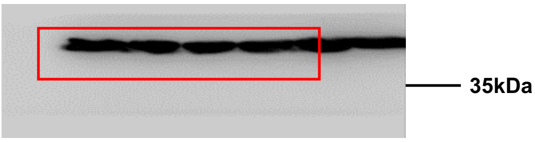

Corresponds to Fig. S5A, first row.

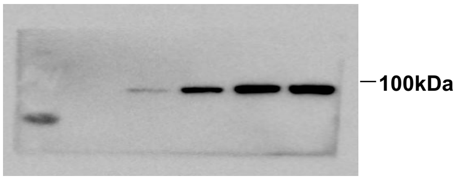

Corresponds to Fig. S5A, second row.

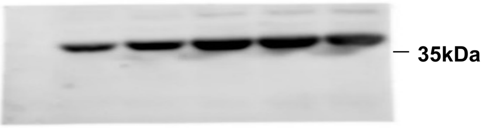

Corresponds to Fig. S5A, third row.

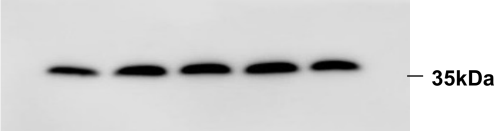

Corresponds to Fig. S3B, first row.

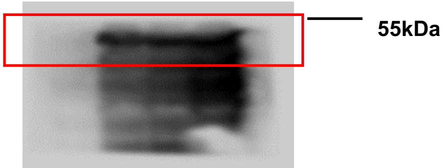

Corresponds to Fig. S3B, second row.

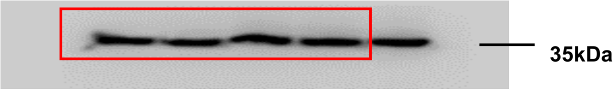

Corresponds to Fig. S3B, third row.

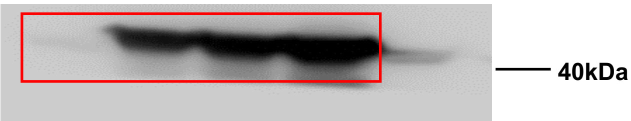

Corresponds to Fig. S3B, fourth row.

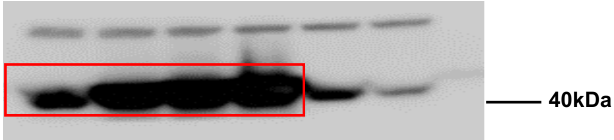

Corresponds to Fig. S3B, fifth row.

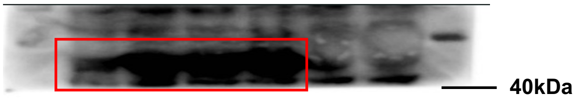

Corresponds to Fig. S3B, sixth row.

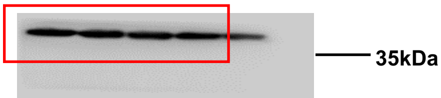

Corresponds to Fig. S5B, first row.

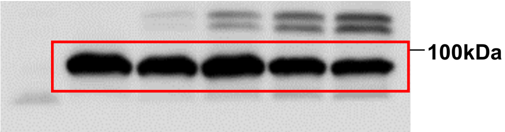

Corresponds to Fig. S5B, second row.

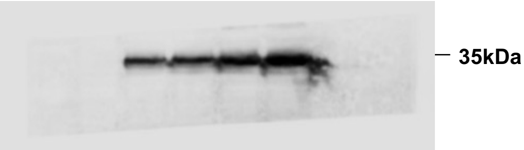

Corresponds to Fig. S5B, third row.

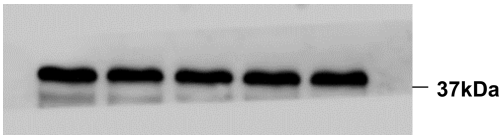

Uncropped whole blots of figures used in this study

Corresponds to Fig S6, first row, first column.

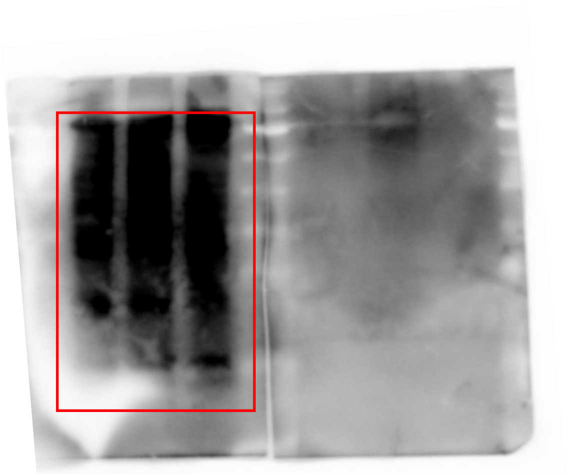

Corresponds to Fig S6, first row, second column.

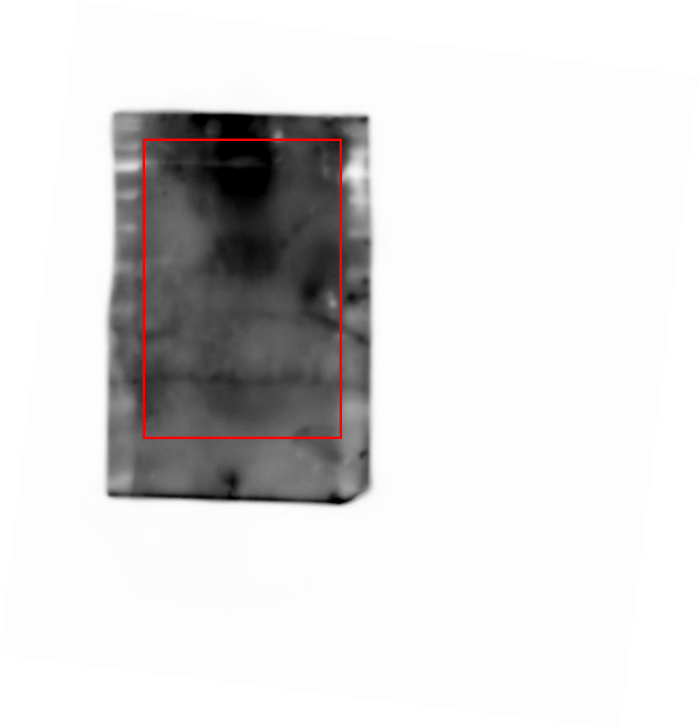

Corresponds to Fig S6, second row.

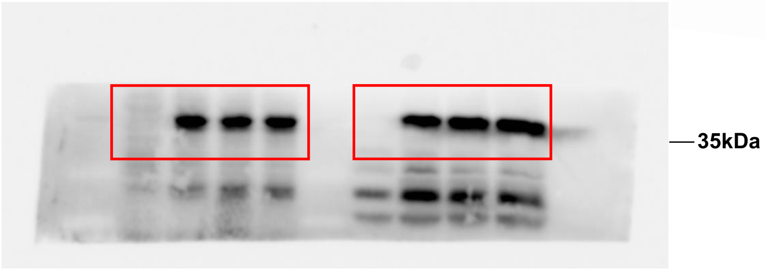

Supplement: Supplementary file 12 — Supplementary Figure S7 [file 41419_2022_5152_MOESM12_ESM.pdf]
